# PNSC928, a plant-derived compound, specifically disrupts CtBP2-p300 interaction and reduces inflammation in mice with acute respiratory distress syndrome

**DOI:** 10.1186/s13062-024-00491-0

**Published:** 2024-06-21

**Authors:** Fan Li, Wenqing Yan, Weihua Dong, Zhiping Chen, Zhi Chen

**Affiliations:** 1 Department of Pulmonary and Critical Care Medicine, The Second Affiliated Hospital of Nanchang University, Nanchang, Jiangxi 330006 China; 2 Department of Critical Care Medicine, Tongji Hospital, School of Medicine, Tongji University, No. 389 Xincun Road, Shanghai, Shanghai 200065 China; 3 Department of Emergency, The First Affiliated Hospital of Nanchang Medical College, Nanchang, Jiangxi 330006 China; 4 Present Address: Department of Emergency, Jiangxi Provincial People’s Hospital, No. 92, Aiguo Road, Donghu District, Nanchang, Jiangxi 330006 China

**Keywords:** PNSC928, ARDS, CtBP2, p300, Proinflammatory cytokine genes

## Abstract

**Background:**

Prior research has highlighted the involvement of a transcriptional complex comprising C-terminal binding protein 2 (CtBP2), histone acetyltransferase p300, and nuclear factor kappa B (NF-κB) in the transactivation of proinflammatory cytokine genes, contributing to inflammation in mice with acute respiratory distress syndrome (ARDS). Nonetheless, it remains uncertain whether the therapeutic targeting of the CtBP2-p300-NF-κB complex holds potential for ARDS suppression.

**Methods:**

An ARDS mouse model was established using lipopolysaccharide (LPS) exposure. RNA-Sequencing (RNA-Seq) was performed on ARDS mice and LPS-treated cells with CtBP2, p300, and p65 knockdown. Small molecules inhibiting the CtBP2-p300 interaction were identified through AlphaScreen. Gene and protein expression levels were quantified using RT-qPCR and immunoblots. Tissue damage was assessed via histological staining.

**Key findings:**

We elucidated the specific role of the CtBP2-p300-NF-κB complex in proinflammatory gene regulation. RNA-seq analysis in LPS-challenged ARDS mice and LPS-treated CtBP2-knockdown (CtBP2^KD^), p300^KD^, and p65^KD^ cells revealed its significant impact on proinflammatory genes with minimal effects on other NF-κB targets. Commercial inhibitors for CtBP2, p300, or NF-κB exhibited moderate cytotoxicity in vitro and in vivo, affecting both proinflammatory genes and other targets. We identified a potent inhibitor, PNSC928, for the CtBP2-p300 interaction using AlphaScreen. PNSC928 treatment hindered the assembly of the CtBP2-p300-NF-κB complex, substantially downregulating proinflammatory cytokine gene expression without observable cytotoxicity in normal cells. In vivo administration of PNSC928 significantly reduced CtBP2-driven proinflammatory gene expression in ARDS mice, alleviating inflammation and lung injury, ultimately improving ARDS prognosis.

**Conclusion:**

Our results position PNSC928 as a promising therapeutic candidate to specifically target the CtBP2-p300 interaction and mitigate inflammation in ARDS management.

**Supplementary Information:**

The online version contains supplementary material available at 10.1186/s13062-024-00491-0.

## Introduction

Acute Respiratory Distress Syndrome (ARDS) is a critical pulmonary disorder marked by abrupt respiratory failure due to pronounced lung inflammation [1, 2]. This condition manifests when fluid inundates the alveoli, the minute air sacs facilitating gas exchange, resulting in significantly reduced oxygen levels in the blood [1, 2]. Such fluid buildup stems from an intensified inflammatory reaction, often induced by infections, trauma, or specific medical procedures [1, 2]. The inflammation in ARDS originates from the release of proinflammatory cytokines and other agents that compromise the integrity of the alveolar-capillary barrier, causing fluid seepage and hindering oxygenation [3, 4]. The nexus between inflammation and ARDS is vital, as persistent inflammation can escalate to lung tissue deterioration, fibrosis, and potentially lethal outcomes [3, 4]. Deciphering the inflammatory mechanisms in ARDS is essential to craft efficacious therapeutic strategies, aiming to temper the inflammatory cascade.

In managing ARDS, medicinal therapy primarily emphasizes respiratory support and treating underlying causes [5, 6]. While no single drug universally addresses ARDS, several are integral to its treatment [5, 6]. Corticosteroids like methylprednisolone, prized for their anti-inflammatory effects, are explored to mitigate the severe inflammation characteristic of ARDS [7, 8]. However, the deployment of corticosteroids is debated, necessitating a careful balance of benefits and risks [7, 8]. In the initial phases of acute ARDS, neuromuscular blocking agents, such as cisatracurium, can be employed to enhance oxygenation, improve lung adaptability, and minimize ventilator-associated lung damage [9, 10]. If infections trigger ARDS, targeted antibiotics or antiviral treatments are indispensable [11]. Inhaled nitric oxide, a pulmonary vasodilator, can temporarily boost oxygenation, though its effect on mortality is negligible [12]. Emerging research probes the potential of therapies like surfactant replacement, antioxidants, and immune modulators, but definitive evidence of their efficacy remains forthcoming [1, 2, 13]. Personalized therapeutic strategies, paired with ongoing research, are essential to refine the pharmacologic approach to ARDS [14].

In eukaryotic systems, gene regulation is regulated by transcription factors and various transcriptional modulators, encompassing entities like histone deacetylases, histone acetyltransferases, and corepressors/coactivators [15]. C-terminal binding proteins (CtBPs), notably CtBP1 and CtBP2, function as dual-faceted transcriptional regulators, serving as both corepressors and coactivators in varying biological contexts [15]. Intriguingly, while CtBP1’s involvement remains unasserted, CtBP2 has been directly linked with the molecular underpinnings of ARDS [16, 17]. Research indicates that CtBP2 forms a complex with the histone acetyltransferase p300 and nuclear factor kappa B (NF-κB) [16]. This assembly is instrumental in the transactivation of specific proinflammatory cytokine genes, including interleukin (IL)-1β, IL-6, IL-15, IL-18, and tumor necrosis factor-alpha (TNF-α) [16]. The upregulation of these genes intensifies lung inflammation, a defining characteristic of ARDS [16]. Delving into the functions of CtBP2 and its intertwined molecular pathways could unveil promising therapeutic avenues for modulating inflammation, ultimately enhancing prognosis for ARDS patients.

Multiple genes are intricately regulated by CtBP2, p300, and NF-κB. Specifically, CtBPs are involved in modulating genes implicated in tumor suppression and cell cycle progression, such as E-cadherin (also known as cadherin 1, CDH1), cyclin-dependent kinase inhibitor 1 A (CDKN1A), and CDKN2A [18]. Additionally, they oversee developmental and differentiation processes by regulating genes like Chordin (CHRD) and Paired box 6 (PAX6) [19], as well as apoptosis through genes such as BCL2 Associated X (Bax) and BCL2 binding component 3 (BBC3) [18]. p300, in contrast, orchestrates the inflammatory response in synovial fibroblasts by modulating a plethora of genes, including but not limited to Nucleotide-binding oligomerization domain containing 2 (NOD2), C-C motif chemokine ligand 2 (CCL2), C-X-C motif chemokine ligand 10 (CXCL10), CXCL12, STAT1 (Signal transducer and activator of transcription 1), and MMP1 (Matrix metallopeptidase 1) [20–22]. NF-κB is known to influence several hundred genes. These encompass inflammatory cytokines and chemokines (e.g., IL-1, IL6, IL8, TNF-α, and MCP-1), cell adhesion molecules (e.g., ICAM1 and VCAM1), growth factors (e.g., GM-CSF and VEGF), enzymes (e.g., COX2 and iNOS), and immunoregulatory genes (e.g., IL2 and IL12) [23]. While there are several inhibitors targeting CtBP2 (e.g., MTOB and NSC95397) [15], p300 (e.g., C646 and A-485) [24], and NF-κB (e.g., TPCA1 and BOT64) [25], their specificity remains a concern. Using these inhibitors to mitigate inflammation in ARDS induced by inflammatory agents could lead to unintended side effects. This is due to their potential to alter the expression of a broad spectrum of target genes, not just proinflammatory cytokine genes.

Here, we used RNA-Seq to study gene changes in mice with lipopolysaccharides (LPS)-induced ARDS and LPS-treated CtBP2-knockdown (CtBP2^KD^), p300^KD^, and p65^KD^ cells, emphasizing the CtBP2-p300-NF-κB specificity in proinflammatory gene regulation. Inhibiting CtBP2, p300, or NF-κB improved inflammatory symptoms in ARDS mice but caused side effects from altered other target gene expression. Using the Amplified Luminescent Proximity Homogeneous Assay (AlphaScreen), we identified PNSC928 from a plant-derived chemical library that disrupted the CtBP2-p300 interaction. Our tests showed that PNSC928 specifically inhibits proinflammatory genes, benefiting ARDS mice, without significantly affecting other CtBP2, p300, and NF-κB target genes.

## Materials and methods

### Animal experiments

All animal experiments were performed following a protocol reviewed and approved by the Institutional Animal Care and Use Committee at Jiangxi Provincial People’s Hospital. We procured C57BL/6 mice from Charles River Laboratories (Beijing, China) and maintained them in specific pathogen-free (SPF) conditions, with a 12-hour light/dark cycle and free access to food and sterile water. We established the ARDS mouse model by administering LPS via tracheal intubation, following an established protocol [26]. Briefly, mice (eight-week-old, half male and half female, 22–25 g) were anesthetized using isoflurane inhalation and randomly classified into Control and ARDS groups. Control mice received 50 µL sterile saline, while ARDS mice were given 25 µg LPS (Sigma-Aldrich, Shanghai, China; #LPS25) in 50 µL sterile saline. Mice were immediately positioned upright and rotated to evenly distribute the LPS within the lungs.

To assess the impact of CtBP2, p300, NF-κB inhibitors, and PNSC928 on ARDS progression, mice were further categorized post-4 h LPS administration into different groups: ARDS, ARDS + MTOB (700 mg/kg; MedChemExpress; Monmouth Junction, NJ; #HY-135,046), ARDS + NSC95397 (1.5 mg/kg; MedChemExpress; #HY-108,543), ARDS + C646 (10 mg/kg; MedChemExpress; #HY-13,823), ARDS + A-485 (50 mg/kg; MedChemExpress; #HY-107,455), ARDS + TPCA1 (10 mg/kg; MedChemExpress; #HY-10,074), ARDS + BOT64 (50 mg/kg; MedChemExpress; #HY-136,741), and ARDS + PNSC928 (20 mg/kg). Mice received these compounds daily for two days. Body weight and temperature were recorded every 24 h. At 48 h post-LPS challenge, mice were sacrificed, and blood samples and lung tissues were harvested.

To collect bronchoalveolar lavage fluid (BALF), the Control, ARDS, and ARDS + PNSC928 groups of mice (*n* = 10 for each group) were anesthetized with isoflurane inhalation in accordance with institutional guidelines. Mice were placed on their backs, and a small incision was made at the base of the neck to expose the trachea. A flexible catheter was inserted into the trachea and secured with a suture. A syringe filled with sterile phosphate-buffered saline (PBS) (Thermo Fisher, Shanghai, China; #10,010,023) was attached to the catheter, and 1.5 mL of PBS was gently instilled into the right lungs. After allowing the fluid to sit for 10 s, it was aspirated back into the syringe. This process was repeated three times to ensure thorough lavage. The collected BALF was kept on ice in a sterile tube, then centrifuged at 1,000 × g for 10 min at 4 °C to pellet the cells. The supernatant was stored at -80 °C for further analyses.

### Cells, cell culture, and transfection

Mouse pulmonary alveolar epithelial cells (MPAEpiC) were sourced from ScienCell Research Laboratories (Carlsbad, CA, USA; #M3200-57). The mouse macrophage cell line RAW 264.7 (#TIB-71), the mouse liver hepatocyte cell line FL83B (#CRL-2390), and the mouse kidney epithelial-like cell line TKPTS (#CRL-3361) were sourced from the American Type Culture Collection (ATCC) (Manassas, VA, USA). Additionally, the mouse cardiac muscle cell line HL-1 (SCC065) was acquired from Sigma-Aldrich. All cell lines were maintained in Dulbecco’s Modified Eagle Medium (DMEM) (Thermo Fisher; #A1048901) fortified with 10% fetal bovine serum (FBS) (Thermo Fisher; #A5256701) and a combination of 100 U/mL penicillin and streptomycin (Thermo Fisher; #15,140,122). Cells were cultured in a 37 °C incubator with 5% CO_2_, and the medium was changed every 2–3 days. For targeted gene manipulation, cells were transfected with gene-specific short hairpin RNAs (shRNAs) (Table S1) or plasmids (Table S2). Transfection was facilitated using the Lipofectamine 3000 Transfection Reagent (Thermo Fisher; #L3000001) as per the manufacturer’s recommended protocol.

### Cell treatments

For LPS treatment, different knockdown (KD) cell lines—Control^KD^, CtBP2^KD^, p300^KD^, and p65^KD^, all derived from MPAEpiC—were seeded in 12-well plates and cultured at 37 °C until reaching 80% confluence. The cells were then treated with 200 ng/mL of LPS for 6 h, washed three times with PBS, and subjected to the required experiments. For experiments involving inhibitors of CtBP2, p300, and NF-κB, as well as the compound PNSC928, MPAEpiC, RAW264.7, FL83B, TKPTS, and HL-1 cells were seeded in 12-well plates and cultured at 37 °C to reach 80% confluence. Cells were then cotreated with 200 ng/mL LPS along with varying concentrations of the following chemicals: MTOB (0, 5, 10, or 20 mM), NSC95397 (0, 10, 20, or 40 µM), C646 (0, 3, 6, or 12 µM), A-485 (0, 5, 10, or 20 nM), TPCA1 (0, 150, 300, or 600 nM), BOT64 (0, 1, 2, or 4 µM), or PNSC928 (0, 11.8, 23.6, or 47.2 µM). After 6 h of treatment, cells were washed three times with PBS before proceeding with further experiments.

### Determination of serum and BALF proinflammatory cytokine levels

Whole blood samples were allowed to stand undisturbed at room temperature for 15 min before being centrifuged at 1,000 × g for 10 min. The supernatant serum and BALF were used to measure concentrations of IL-1β, IL-6, IL-15, IL-18, and TNF-α with specific enzyme-linked immunosorbent assay (ELISA) kits as per manufacturer guidelines. The ELISA kits used were: IL-1β (Thermo Fisher; #KMC0011), IL-6 (Thermo Fisher; #KMC0061), IL-15 (Abcam; Shanghai, China; #ab275898), IL-18 (Thermo Fisher; #BMS618-3), and TNF-α (Thermo Fisher; #BMS607-3). Total BALF protein levels were measured using the BCA Protein Assay Kit (Abcam; #ab102536) according to the manufacturer’s protocol.

### Evaluation of liver and kidney functions

Serum activities or levels of alanine aminotransferase (ALT), aspartate aminotransferase (AST), alkaline phosphatase (ALP), blood urea nitrogen (BUN), and creatinine were measured using commercially available assay kits, including the ALT Activity Assay Kit (Abcam; #ab105134), AST Activity Assay Kit (Abcam; #ab105135), ALP Assay Kit (Abcam; #ab83369), BUN Colorimetric Detection Kit (Thermo Fisher; #EIABUN), and Creatinine Assay Kit (Abcam; #ab65340). All experimental procedures were conducted according to the respective manufacturer’s protocols.

### Myeloperoxidase (MPO) activity assay in lung tissues

MPO activity was determined according to a previously established protocol [27]. Briefly, lung tissues (50 mg per mouse) were washed with ice-cold PBS to remove residual blood, then homogenized in a buffer containing 2.5 g of hexadecyltrimethylammonium bromide (Sigma-Aldrich; # H5882) dissolved in 500 mL of PBS. The tissue homogenate was kept on ice for 30 min before centrifugation at 16,000 × g for 10 min at 4 °C. The supernatant was used to measure MPO activity with the Myeloperoxidase Activity Assay Kit (Abcam; #ab105136) according to the manufacturer’s method.

### Determination of lung wet/dry weight ratio

The right upper lobe of the lung was excised, rinsed with PBS, blotted dry, and weighed to determine the “wet” weight. The lung tissue was then placed in an oven set at 70 °C for 72 h, after which it was re-weighed to obtain the “dry” weight. The wet/dry weight ratio was calculated to assess the degree of pulmonary edema.

### RNA-sequence (RNA-Seq) analysis

Total RNA was extracted utilizing the NucleoSpin RNA kit (TaKaRa, Beijing, China; #740984.50) as directed by the manufacturer. Subsequent analysis of RNA concentration and integrity was carried out with the NanoDrop spectrophotometer. A quantity of 15 µg RNA was allocated for the enrichment of poly(A)-tailed mRNA molecules, subsequent amplification, and cDNA dual-strand synthesis in alignment with the TruSeq RNA Sample Prep protocol by Illumina. RNA sequencing was performed on an Illumina NovaSeq 6000 sequencer, with data analysis entrusted to GenomeScan (www.genomescan.nl). This comprehensive procedure encompassed data quality assurance, adapter sequence removal, short-read alignment, and feature quantification. The integrity of library preparation was verified by evaluating ribosomal content, while contamination checks ensured sample and barcode purity. Initial dataset quality was ascertained using FastQC v0.34 and FastQA. Reads underwent adapter sequence trimming via Trimmomatic v0.30 before alignment against the mouse reference genome GRCm38 (patch 6). Post-sequencing analyses were conducted on the BioJupies platform (https://amp.pharm.mssm.edu/biojupies/) and the DeSeq2 package within R. Expression data for genes in murine lungs were sourced from the NCBI GEO database (GSE151674) through matrix file downloads. Functional, pathway, and gene ontology (GO) enrichment analyses were conducted utilizing the Database for Annotation, Visualization, and Integrated Discovery (DAVID).

### Reverse transcription-quantitative polymerase chain reaction (RT-qPCR)

RNA was isolated from both cultured cells and mouse lung tissues utilizing the TRIzol Reagent (Invitrogen; Shanghai, China; #15,596,018) in adherence to the manufacturer’s protocol. For each sample, 500 ng of RNA was employed for the synthesis of complementary DNA (cDNA) leveraging the High-Capacity cDNA Reverse Transcription Kit (Thermo Fisher; #4,368,814). Gene expression quantification was carried out using the SYBR GreenER qPCR SuperMix Universal (Thermo Fisher; #1176202K) in combination with the specific primers delineated in Table S3. Subsequent analysis normalized the expression levels to β-Actin, utilizing the 2^−ΔΔCt^ method.

### Total protein extraction and immunoblots

Cellular and mouse lung tissue lysates were prepared using the RIPA buffer (Thermo Fisher; #PI89900) supplemented with 1 × complete protease inhibitor cocktail (Thermo Fisher; #87,785). Following a 10-minute centrifugation at 13,000 g, proteins (50 µg) from the cleared lysate were resolved via 10% SDS-PAGE. These separated proteins were electroblotted onto PVDF membranes (Thermo Fisher; #88,585). The membranes were blocked with 5% non-fat milk (Sigma-Aldrich; #M7409) in phosphate-buffered saline with Tween 20 (PBST) (Abcam; #ab64204) for 1 h at room temperature. After blocking, the membranes were incubated with the specified primary antibodies (Table S4). Following the primary antibody incubation, the PVDF membranes were washed four times with PBST, each wash lasting 10 min at room temperature. Next, the membranes were incubated with horseradish peroxidase-linked secondary antibodies (Table S4) for 1 h at room temperature. After secondary antibody incubation, the membranes were washed again four times with PBST, each wash for 10 min, to remove unbound antibodies. Visualization of protein bands was facilitated by the Pierce ECL Western Blotting Substrate (Thermo Fisher; #32,106). Densitometric analysis of the protein bands was performed using Image J software (version 1.53t, National Institutes of Health, USA), with results being normalized against the loading control.

### Separation of nuclear and cytoplasmic proteins

Nuclear and cytoplasmic proteins were extracted using NE-PER Nuclear and Cytoplasmic Extraction Reagents (Thermo Fisher; #78,833) per the manufacturer’s instructions. Briefly, 100 mg of lung tissue was washed twice with PBS and then cut into small pieces. The tissue was homogenized in 1 mL of Cytoplasmic Extraction Reagent I (CER I) containing a 1 × complete protease inhibitor cocktail using a KIMBLE Dounce tissue grinder (Sigma-Aldrich; #D9063). The homogenate was vortexed at the highest setting for 15 s and incubated on ice for 10 min. Next, 55 µL of ice-cold CER II was added, vortexed for 5 s, incubated on ice for 1 min, and then vortexed again for 5 s. The sample was centrifuged at 16,000 × g for 15 min. The supernatant containing the cytoplasmic fraction was collected and stored at -80 °C. The nuclear pellet was resuspended in 500 µL of ice-cold Nuclear Extraction Reagent (NER), vortexed for 15 s, and kept on ice, with additional 15-second vortexing every 10 min for 40 min. After centrifugation at 16,000 × g for 10 min, the nuclear supernatant was collected and stored at -80 °C until further use.

### Immunoprecipitation assay

The extracted nuclear fractions (300 µL each) from different groups of lung tissues were incubated with 0.5 µL of anti-CtBP2 antibody at 4 °C for 12 h with gentle agitation. Next, 20 µL of protein A agarose beads (Thermo Fisher; #20,333) were added to the nuclear fractions to facilitate immunoprecipitation, followed by incubation at 4 °C with rotary agitation for 4 h. The tubes were then centrifuged to pellet the beads, and the supernatant was discarded. To minimize non-specific binding, the beads were washed 5 times with a buffer containing 10 mM Tris (pH 7.4), 1 mM EDTA, 1 mM EGTA (pH 8.0), 150 mM NaCl, 1% Triton X-100, 0.2 mM sodium orthovanadate, and a 1 × complete protease inhibitor cocktail. For each wash, the beads were gently mixed with the washing buffer, then centrifuged at 4 °C, and the supernatant was discarded. After the washing steps, 50 µL of 2 × SDS buffer with DTT was added to the pelleted beads to elute the bound proteins. The samples were then boiled at 95 °C for 5 min. Subsequently, the eluted proteins were analyzed by Western blot to determine their identity and levels.

### Histology assay

Lung, heart, liver, and kidney tissues were collected from various groups of mice, including ARDS, ARDS + MTOB, ARDS + NSC95397, ARDS + C646, ARDS + A-485, ARDS + TPCA1, ARDS + BOT64, and ARDS + PNSC928. The tissues were fixed in 10% neutral-buffered formalin (Sigma-Aldrich; #HT501128) for 48 h, then dehydrated through a graded ethanol series (70%, 80%, 90%, and 100%, for 30 min each). The dehydrated tissues were embedded in paraffin blocks and allowed to solidify. The paraffin blocks were sectioned at 5 μm, with the sections floated on a water bath to remove wrinkles before being transferred onto glass slides. The slides were dried, dewaxed with xylene, and rehydrated through a reverse ethanol series (100%, 90%, 80%, and 70%, for 30 min each), followed by a final rinse in distilled water. The rehydrated sections were stained using the Hematoxylin and Eosin Kit (Abcam; #ab245880) according to the manufacturer’s guidelines. After staining, the sections were dehydrated through a graded ethanol series (70%, 80%, 90%, and 100%, for 30 min each), then cleared with xylene. The sections were mounted with a mounting medium (Abcam; #ab64230) and imaged using a Nikon light microscope for tissue morphology.

Lung injury was scored according to a previously established protocol [28], with four assessment criteria: alveolar congestion, hemorrhage, infiltration or aggregation of neutrophils in airspaces or vessel walls, and the thickness of alveolar walls or membrane formation. The severity was graded on a scale from 0 to 4 (0, normal; 1, mild; 2, moderate; 3, severe; 4, intense).

### AlphaScreen

Plasmids pGEX-6P-1-p300^BRD^ and pET-28a-CtBP2 (Table S2) were transformed into BL21 competent cells. Upon 1 mM isopropyl β-d-1-thiogalactopyranoside (IPTG) induction, GST-p300BRD and His-CtBP2 proteins were isolated via Glutathione Agarose Beads (Thermo Fisher; #16,100) and Ni-NTA Agarose (Thermo Fisher; #R90115), respectively. A comprehensive small molecule library of 4313 compounds derived from various botanical sources, including Lonicera japonica, Forsythia suspense, Houttuynia cordata, Coptis chinensis, Phellodendron, Pulsatilla chinensis, Lygodium japonicum, Plantago asiatica, and Portulaca olerace, was analyzed through mass spectrometry. The CtBP2-p300^BRD^ interaction-disrupting molecules were pinpointed utilizing the AlphaScreen detection kit (PerkinElmer; #6,760,603 M), following the manufacturer’s directives. In a concise methodology, both proteins at a concentration of 100 nM were combined with 10 µL of AlphaScreen donor and acceptor beads. Post addition of 5 µM from each botanical molecule and a 2-hour incubation at 25 °C, the resultant AlphaScreen signals from 96-well plates (PerkinElmer, #6,008,350) were deciphered with an Envision Multilabel Reader (PerkinElmer, #2105-0010). Compounds exhibiting signals beneath 5000 were earmarked as prospective disruptors.

### Statistical analysis

All experiments were conducted independently a minimum of three times. In animal experiments, “n” represents the number of animals used. For cell culture experiments, the ‘n’ values indicate the number of independent experiments conducted. Each independent experiment included triplicate cultured wells. Data are expressed as mean ± standard deviation (SD) from a representative repeat. For comparisons between the two groups, a two-tailed Student’s t-test was employed. When assessing three or more groups, one-way ANOVA followed by Tukey’s post-hoc test was utilized. Statistical significance was denoted as *P < 0.05, **P < 0.01, and ***P < 0.001.

## Results

### Predominant induction of proinflammatory cytokine genes in LPS-challenged ARDS mice

**Fig. 1:**
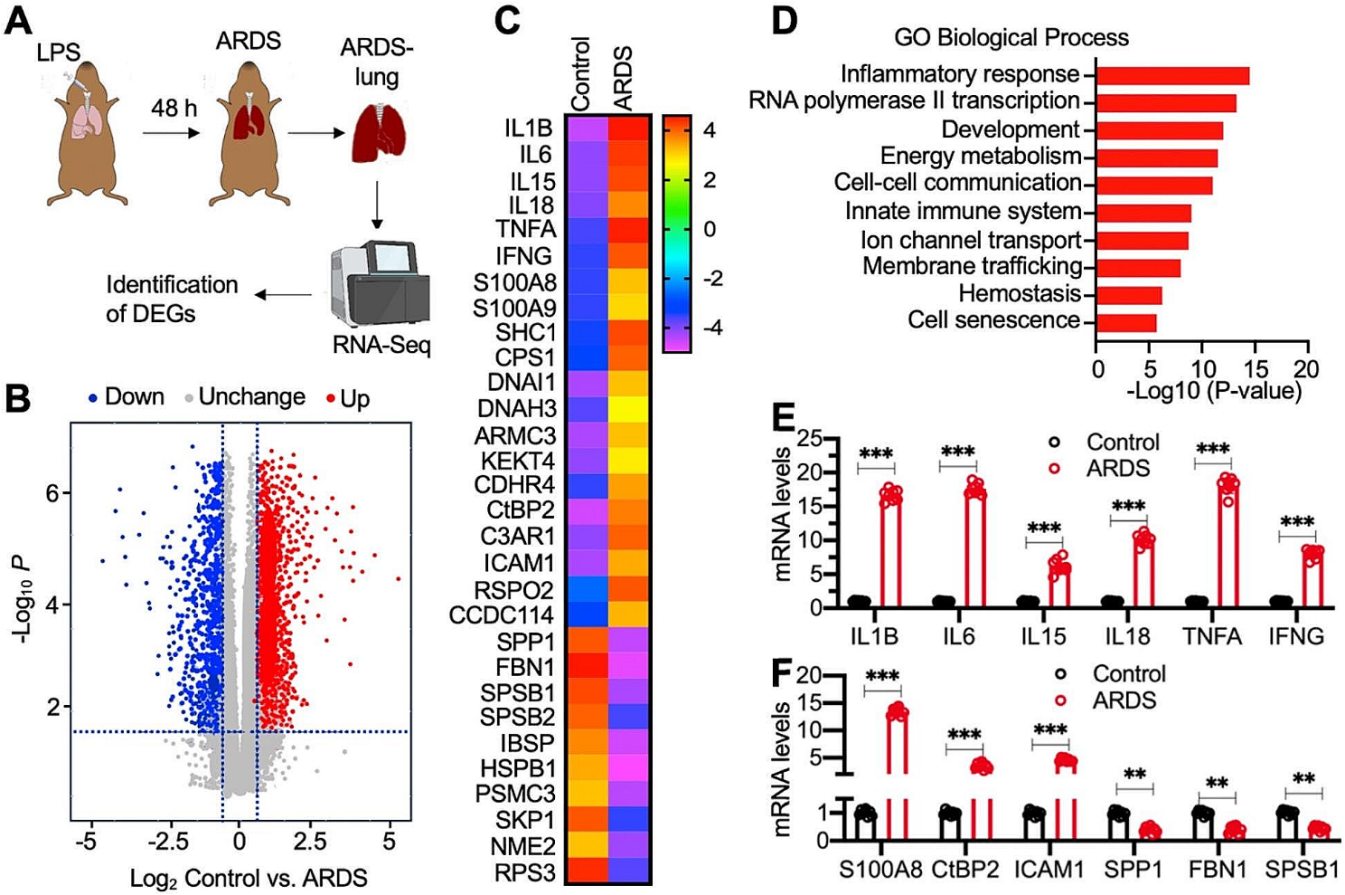
Proinflammatory cytokine genes were predominately upregulated in ARDS mice. **(A)** Experimental design: RNA-Seq analysis to identify differentially expressed genes (DGEs) in ARDS mice. **(B)** Volcano plot displaying RNA-Seq results. **(C)** Heatmap of top 20 upregulated genes and top 10 downregulated genes in ARDS lung tissues. **(D)** Biological processes of DGEs revealed by gene ontology (GO) analysis. **(E and F)** Verification of 12 DGEs by RT-qPCR analysis (*n* = 3). **(E)** IL-1B, IL-6, IL-15, IL-18, TNFA, and IFNG. **(F)** S100A8, CtBP2, ICAM1, SPP1, FBN1, and SPSB1. **P < 0.01; ***P < 0.001

We previously demonstrated that the CtBP2-p300-NF-κB complex modulates proinflammatory cytokine genes, encompassing IL-1B, IL-6, IL-15, IL-18, and TNFA [16]. Despite the extensive downstream targets of CtBP2 and NF-κB and the pivotal role of p300 as a histone modifier, the broader impact on CtBP2 and NF-κB downstream targets post-LPS challenge in vivo remains elusive. To elucidate this, we developed an ARDS mouse model through tracheal intubation and employed RNA-seq to pinpoint differentially expressed genes (DEGs) in the lungs afflicted with ARDS (Fig. 1A). After LPS challenge, we observed that mice showed signs of respiratory distress and a decrease in body weight (Figures S1A and S1B). Additionally, we measured the levels of proinflammatory cytokines in serum and BALF, finding significantly elevated levels of IL-1β, IL-6, IL-15, IL-18, TNF-α, and IFN-γ in ARDS mice (Figures S1C-S1N). The total protein content in BALF, MPO activity in lung tissue, and the lung wet/dry weight ratio in ARDS mice were also significantly elevated (Figures S1O-S1Q). These results indicate that LPS-challenged mice exhibit severe symptoms of ARDS.

We next collected lung tissues from Control and ARDS mice for RNA-Seq analysis. The RNA-Seq data delineated 842 significantly elevated transcripts (fold change > 2) and 706 notably diminished transcripts (fold change < 0.5) in ARDS-afflicted lungs (Fig. 1B). Subsequent Gene Ontology (GO) assessment identified prominent gene clusters, with proinflammatory cytokine genes emerging as the most markedly augmented (Fig. 1C and D, S2, and Table S5). Beyond these, expression alterations were noted in established NF-κB downstream targets such as ICAM1 and SPP1 (Secreted Phosphoprotein 1) (Fig. 1C). Authenticating our findings using lung RNA samples from both control and LPS-challenged ARDS mice, a pronounced amplification of proinflammatory cytokine genes [IL-1B, IL-6, IL-15, IL-18, TNFA, and interferon-gamma (IFNG)] was observed (Fig. 1E). Specific inductions included: IL-1B at 18.3 ± 2.1-fold, IL-6 at 19.2 ± 2.7-fold, IL-15 and IL-18 at approximately 5.7 ± 0.54 and 10.2 ± 0.84-fold, respectively, and TNFA and IFNG at 21.4 ± 3.2-fold and 9.3 ± 0.81-fold, respectively (Fig. 1E). Aligned with our earlier findings, the CtBP2 mRNA level surged to nearly 3.7 ± 0.41-fold (Fig. 1F). Furthermore, gene expressions of S100A8 (S100 calcium-binding protein A8), ICAM1, SPP1, FBN1 (Fibrillin 1), and SPSB1 (SPRY domain-containing SOCS box protein 1) were validated, corroborating the RNA-Seq results (Fig. 1F). Collectively, these outcomes propose that although ARDS-afflicted lungs primarily exhibit changes in proinflammatory cytokine genes, select NF-κB target genes are also subtly impacted.

We additionally investigated the expression of the CtBP2-p300-NF-κB complex in both the cytoplasmic and nuclear fractions of lung tissues from ARDS mice. Our findings indicated a significant reduction in the expression levels of CtBP2, p300, and NF-κB subunits (p50 and p65) within the cytoplasm of ARDS mouse lung tissues, concomitant with a pronounced accumulation of these proteins within the nucleus (Figures S3A and S3B). Immunoprecipitation assay highlighted an augmented binding of the nuclear CtBP2 fraction to p300 and NF-κB subunits (p50 and p65) in ARDS lungs relative to the controls (Figure S3C).

### Predominant reduction of proinflammatory cytokine genes in LPS-treated CtBP2^KD^/p300^KD^/p65^KD^ cells

**Fig. 2:**
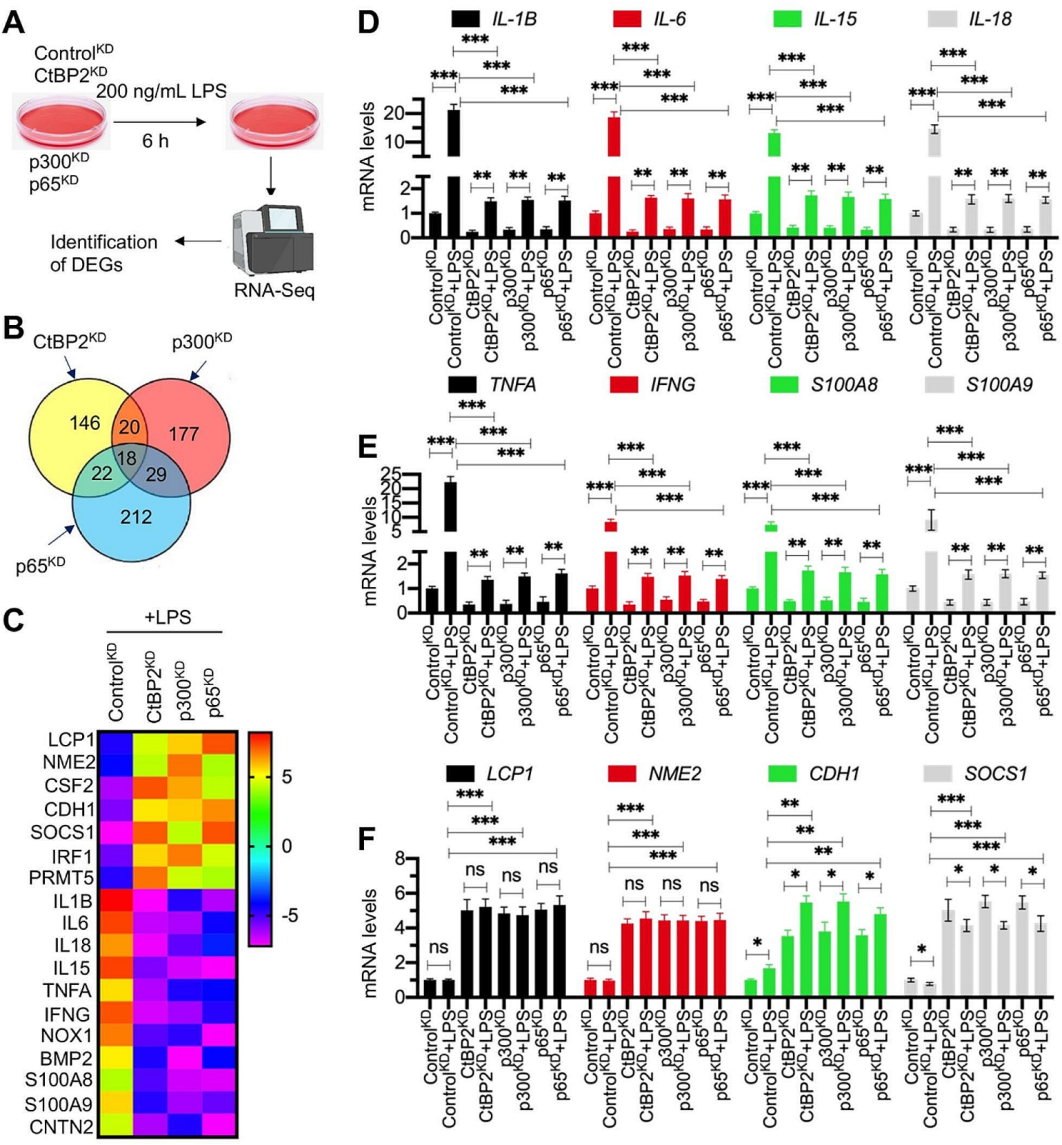
Proinflammatory cytokine genes were downregulated in knockdown cell lines of CtBP2-p300-NF-κB complex members. **(A)** Experimental design: RNA-Seq analysis to DGEs in LPS-challenged Control^KD^, CtBP2^KD^, p300^KD^, and p65^KD^ cells. **(B)** Venn diagram illustrating the altered gene profiles in LPS-challenged Control^KD^, CtBP2^KD^, p300^KD^, and p65^KD^ cells. **(C)** Heatmap displaying the top 7 upregulated genes and top 11 downregulated genes in LPS-challenged Control^KD^, CtBP2^KD^, p300^KD^, and p65^KD^ cells. **(D-F)** Verification of 12 DGEs by RT-qPCR analysis (*n* = 3). **(D)** IL-1B, IL-6, IL-15, and IL-18; **(E)** TNFA, IFNG, S100A8, and S100A9; **(F)** LCP1, NME2, CDH1, and SOCS1. *P < 0.05; **P < 0.01; ***P < 0.001

To delve deeper into whether the DEGs depicted in Fig. 1 were dependent on the CtBP2-p300-NF-κB complex, we generated CtBP2^KD^, p300^KD^, and p65^KD^ cells in MPAEpiC background (Figure S4). These cells were then treated with LPS at 200 ng/mL, and their transcriptomic changes were charted via RNA-seq (Fig. 2A). The resultant data outlined 206, 244, and 281 pronouncedly increased transcripts (fold change > 2) and discernibly decreased transcripts (fold change < 0.5) in the LPS-challenged CtBP2^KD^, p300^KD^, and p65^KD^ cells, respectively (Fig. 2B). Upon juxtaposing the overlapping DEGs across LPS-exposed CtBP2^KD^, p300^KD^, and p65^KD^ cells, a prominent feature was the recurrence of six proinflammatory cytokine genes, including IL-1B, IL-6, IL-15, IL-18, TNFA, and IFNG (Fig. 2C and Table S6). Notably, while various NF-κB target genes emerged in the LPS-treated p65^KD^ cells, their expression didn’t falter upon the nullification of CtBP2 and p300 (Fig. 2C and Table S6).

RT-qPCR analyses highlighted a marked reduction in the expression of inflammatory cytokine genes, including IL-1B, IL-6, IL-8, IL-18, TNFA, and IFNG, in LPS-treated CtBP2^KD^/p300^KD^/p65^KD^ cells (Fig. 2D and E). While in Control^KD^ cells, LPS stimulation led to an elevated expression of these genes by 12–20 fold, this surge was attenuated in CtBP2^KD^/p300^KD^/p65^KD^ cells, with just a 1.3–1.8 fold increase (Fig. 2D and E). We also confirmed the expression levels of six additional DEGs depicted in Fig. 2C: S100A8, S100A9, LCP1, NME2, CDH1, and SOCS1, in LPS-treated CtBP2^KD^/p300^KD^/p65^KD^ cells (Fig. 2E and F). The expression levels of these genes aligned with those observed in the RNA-seq (Fig. 2E and F). This data underscores the pivotal role of the CtBP2-p300-NF-κB complex in modulating the expression of proinflammatory cytokine genes, suggesting that other NF-κB targets remain comparatively unaffected.

### Selective limitations of CtBP2, p300, and NF-κB inhibitors in differentiating proinflammatory cytokine genes from other target genes

Multiple inhibitors targeting CtBP2, p300, and NF-κB have been identified [15, 24, 25]. In previous research, we demonstrated that suppressing CtBP2-p300-NF-κB components hinders the LPS-mediated induction of proinflammatory cytokine genes [16]. Consequently, we hypothesized that blocking CtBP2-p300-NF-κB using their respective inhibitors would mitigate LPS-driven proinflammatory cytokine gene expression, thereby potentially ameliorating the in vivo symptoms in ARDS mice.

To validate this, we co-treated MPAEpiC, RAW264.7, FL83B, TKPTS, and HL-1 cells with LPS and a set of inhibitors for CtBP2 (MTOB and NSC95397), p300 (C646 and A-485), and NF-κB (TPCA1 and BOT64). Post-treatment, cell viability assessments revealed that even at lower doses—MTOB (5 mM), NSC95397 (10 µM), C646 (3 µM), A-485 (5 nM), TPCA1 (150 nM), and BOT64 (1 µM)—there was a notable inhibition of cell growth (Figure S5). These findings suggest that the commercially available inhibitors targeting CtBP2, p300, and NF-κB exhibit extensive inhibitory effects on the proliferation of normal cells, extending beyond MPAEpiC cells.

In addition, we also quantified the expression levels of various genes, categorizing them into proinflammatory cytokines (IL-1B, IL-6, IL-8, IL-15, and TNFA), representative CtBP2 targets (CDH1, CDKN1A, CDKN2A, CHRD, PAX6, and BAX), characteristic p300 targets (NOD2, CCL2, CXCL10, CXCL12, MMP1, and STAT1), and illustrative NF-κB targets (MIP2, CCR5, GM-CSF, VEGF, COX2, and iNOS), in MPAEpiC and RAW264.7 cells cotreated with LPS and inhibitors. RT-qPCR data indicated a pronounced induction in the expression of IL-1B, IL-6, IL-15, IL-18, TNFA, CDKN1A, CDKN2A, CCL2, CXCL10, CXCL12, MMP1, MIP2, CCR5, GM-CSF, VEGF, COX2, and iNOS post-LPS exposure (Figures S6A-S6D and S7A-S7D). In contrast, the expression of CDH1 and BAX was diminished (Figures S6B and S7B). Several genes, including CHRD, PAX6, NOD2, and STAT1, were not affected by the LPS challenge (Figures S6B, S6C, S7B, and S7C). Upon introducing CtBP2 inhibitors MTOB and NSC95397, we observed a dose-dependent reversal in the expression of IL-1B, IL-6, IL-15, IL-18, TNFA, CDH1, CDKN1A, CDKN2A, CHRD, PAX6, and BAX, while the expression of the remaining genes remained relatively unchanged (Figures S6A-S6D and S7A-S7D). Similarly, the p300 inhibitors C646 and A-485 significantly modulated the expression of IL-1B, IL-6, IL-15, IL-18, TNFA, NOD2, CCL2, CXCL10, CXCL12, MMP1, and STAT1, but had no discernible impact on other genes under investigation (Figures S6A-S6D and S7A-S7D). Lastly, the NF-κB inhibitors TPCA1 and BOT64 exhibited a dose-dependent alteration in the expression levels of IL-1B, IL-6, IL-8, IL-15, TNFA, MIP2, CCR5, GM-CSF, VEGF, COX2, and iNOS, without affecting the other studied genes (Figures S6A-S6D and S7A-S7D). These observations suggest that these inhibitors do not specifically discriminate between proinflammatory cytokine genes and other target categories.

### Administering CtBP2, p300, and NF-κB inhibitors in vivo led to adverse effects on C57BL/6 mice

**Fig. 3:**
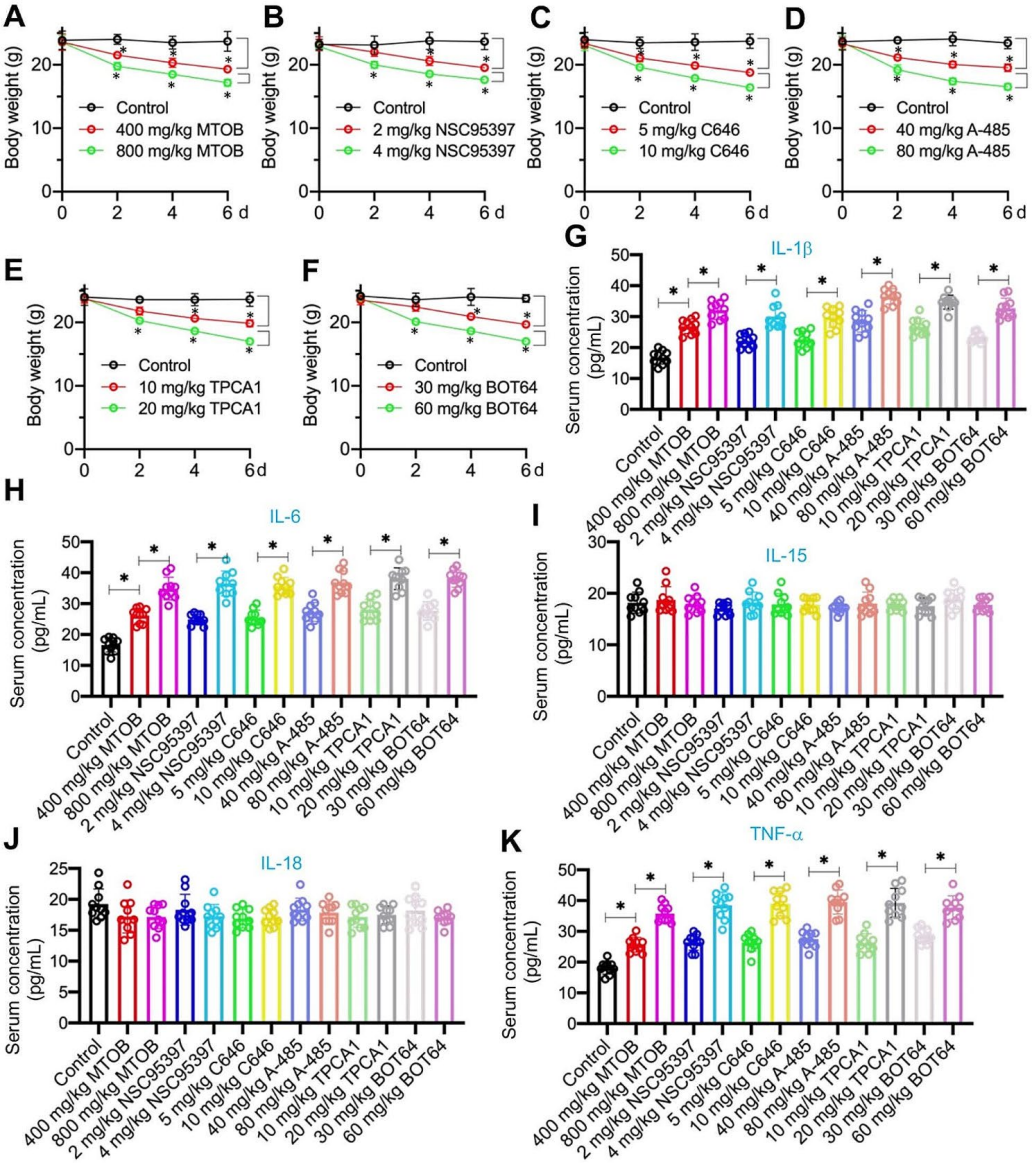
Inhibitors of CtBP2, p300, and NF-κB exhibited cytotoxicity in vivo. C57BL/6 mice were administrated with MTOB (400 and 800 mg/kg), NSC95397 (2 and 4 mg/kg), C646 (5 and 10 mg/kg), A-485 (40 and 80 mg/kg), TPCA1 (10 and 20 mg/kg), BOT64 (30 and 60 mg/kg) for a duration of 6 days (*n* = 10 for each group). **(A-F)** Body weights were measured every two days. **(G-K)** Serum concentrations of different groups of mice. **(G)** IL-1β, **(H)** IL-6, **(I)** IL-15, **(J)** IL-18, and **(K)** TNF-α. *P < 0.05

Further, we evaluated the in vivo effects of six inhibitors on C57BL/6 mice. Upon administering MTOB, NSC95397, C646, A-485, TPCA1, and BOT64, we observed a significant decrease in body weight (Fig. 3A and F). ELISA data highlighted that while all six inhibitors slightly induced serum concentrations of IL-1β, IL-6, and TNF-α but did not affect concentrations of IL-15, IL-18, and IFN-γ (Fig. 3G and K and S8A). We also measured the serum activities of ALT, AST, and ALP, as well as the serum levels of BUN and creatinine. The findings revealed a significant increase in these parameters in all inhibitor-treated groups of mice compared to the control group, demonstrating a dose-dependent effect (Figures S8B-S8D). This suggests the occurrence of liver and kidney damage in all drug-treated groups of mice. H&E staining of organs, including the lung, heart, liver, and kidneys, indicated tissue damage due to these inhibitors (Figure S8E). Based on these findings, the inhibitors may pose potential risks to C57BL/6 mice, suggesting they might not be the optimal choice for treating ARDS to alleviate inflammation.

### Discovery of selective small molecules disrupting the CtBP2-p300^BRD^ interaction using AlphaScreen

**Fig. 4:**
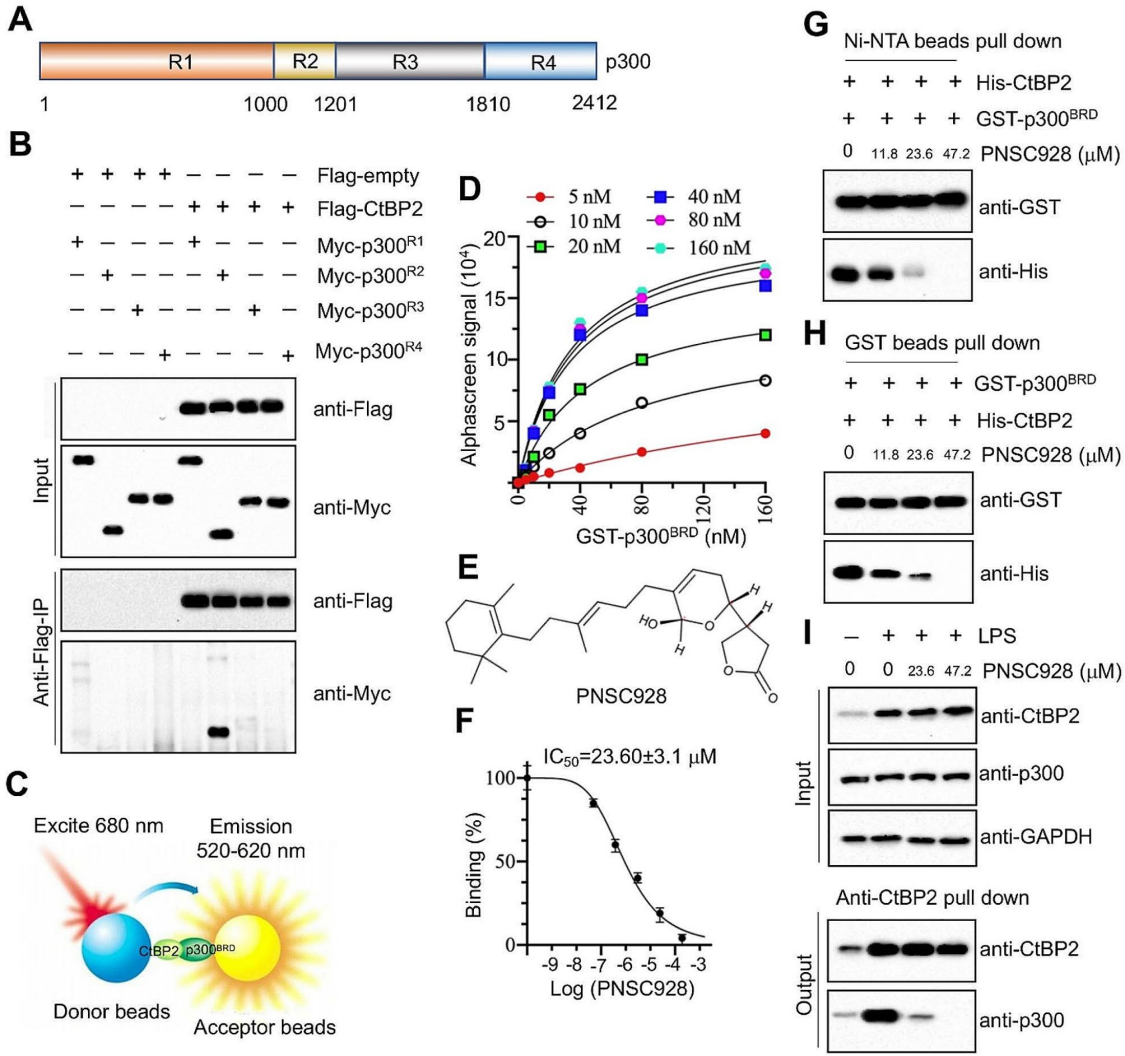
Identification of PNSC928 and determination of its in vitro and in vivo effects on disruption of CtBP2-p300 interaction. **(A)** A schematic representation illustrating four regions of the p300 amino acid sequence. **(B)** Co-IP results. Different combinations of plasmids were cotransfected into MPAEpiC cells. After a 48-hour incubation, cells were lysed and used for immunoprecipitation with anti-Flag agarose. The input and output proteins were probed with anti-Flag and anti-Myc antibodies. *n* = 3. **(C)** A schematic representation illustrating the in vitro AlphaScreen setup. **(D)** AlphaScreen signals for different concentrations of His-CtBP2 and GST-p300^BRD^. **(E)** Chemical structure of PNSC928. **(F)** IC_50_ of PNSC928 disrupting CtBP2-p300^BRD^ interaction. **(G and H)** In vitro pulldown assay in a reaction consisting of His-CtBP2, GST-p300^BRD^, and incremental concentrations of PNSC928 (0, 11.8, 23.6, and 47.2 µM). **(G)** Pulldown with Ni-NTA beads; **(H)** Pulldown with GST beads. **(I)** In vivo immunoprecipitation with anti-CtBP2-coated protein G beads in MPAEpiC cells exposed to varying doses of PNSC928 (0, 11.8, 23.6, and 47.2 µM) over a 6-hour period. *n* = 3

Targeting protein-protein interactions has been recognized as promising avenues for therapeutic development [29]. To enhance the specificity of inhibitors, we focused on screening compounds that specifically target the CtBP2-p300 interaction. We subdivided p300 into four regions: R1 (1-1000 amino acids, aa), R2 (1001–1201 aa), R3 (1202–1810 aa), and R4 (1811–2412 aa) (Fig. 4A). An in vitro co-immunoprecipitation assay highlighted that the R2 region of p300, which contains the PMDLS motif, facilitates its interaction with CtBP2 (Fig. 4B). This R2 region of p300, also termed p300^BRD^, encompasses the Bromo domain.

To probe this interaction, we established an AlphaScreen system that utilized GST-p300^BRD^ and His-CtBP2, binding to donor and acceptor beads, respectively (Fig. 4C). To ascertain optimal protein concentrations for the AlphaScreen interaction, we tested varying concentrations of both GST-p300^BRD^ (0, 5, 10, 20, 40, 80, and 160 nM) and His-CtBP2 (0, 5, 10, 20, 40, 80, and 160 nM). A peak interaction was observed using 80 nM GST-p300^BRD^ in conjunction with 40 nM His-CtBP2 (Fig. 4D). Leveraging these concentrations, we initiated high-throughput screening by individually introducing compounds from a plant-derived chemical library containing 4313 compounds. A standout compound, PNSC928, markedly reduced the Alpha signal from 17,500 to 489 and its chemical structure is presented in Fig. 4E. Subsequent analysis pinpointed PNSC928 as a potent inhibitor of the CtBP2-p300^BRD^ interaction, boasting an IC_50_ value of 23.60 ± 3.1 µM (Fig. 4F). In vitro assays assessed the effects of PNSC928 on the CtBP2-p300^BRD^ interaction. A pulldown assay using varying concentrations of PNSC928 (0, 11.8, 23.6, and 47.2 µM) alongside 80 nM GST-p300^BRD^ and 40 nM His-CtBP2 demonstrated that PNSC928 disrupts the CtBP2-p300^BRD^ interaction in a dose-dependent manner (Fig. 4G and H). Similarly, an immunoprecipitation assay in MPAEpiC cells, co-treated with LPS and PNSC928, indicated a dose-dependent decrease in p300^BRD^ levels pulled down by CtBP2 (Fig. 4I). Furthermore, we assessed the impact of combined LPS and PNSC928 treatment on the expression of key molecules in the NF-κB signaling pathway. Our findings revealed that LPS treatment alone significantly upregulated the phosphorylation levels of IKKα (IκB kinase alpha) and IκBα (Inhibitor of kappa B alpha), while downregulating the protein levels of IκBα and promoting the nuclear translocation of p65 and p50 (Figures S9A and S9B). Interestingly, supplementation of PNSC928 did not alter the effects of LPS on the expression of these proteins (Figures S9A and S9B). These observations suggest that PNSC928 may possess a specific inhibitory effect on the CtBP2-p300 interaction.

**PNSC928 exhibited weak cytotoxicity while maintaining a high specificity in inhibiting the expression of proinflammatory cytokine genes**.

**Fig. 5:**
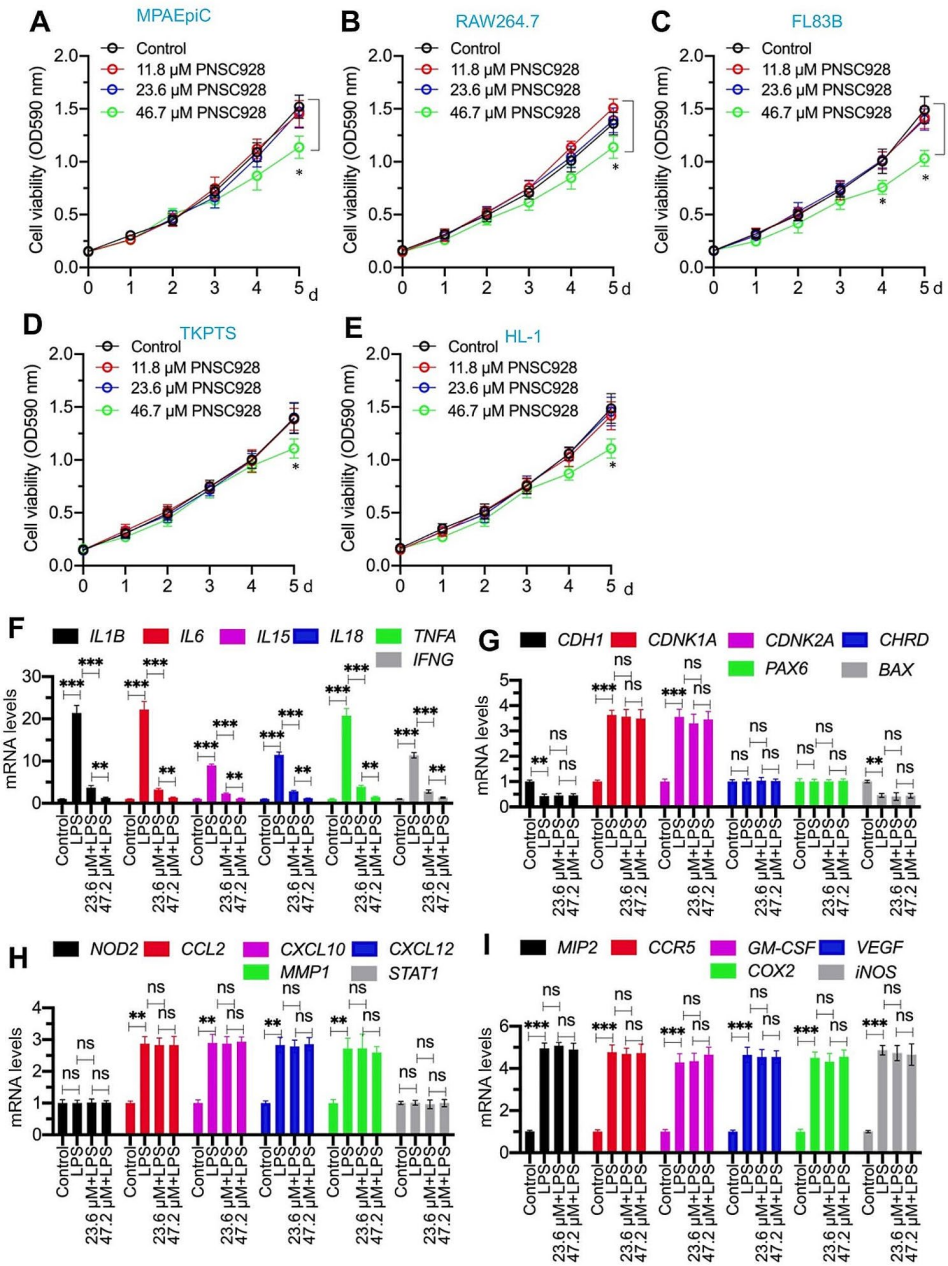
Determination of PNSC928 cytotoxicity and evaluation of PNSC928 effects on the expression levels of CtBP2/p300/NF-κB target genes. **(A-E)** Cell viability assessment. Five cell lines, including MPAEpiC **(A)**, RAW264.7 **(B)**, FL83B **(C)**, TKPTS **(D)**, and HL-1 **(E)** were treated with varying concentrations of PNSC928 (0, 11.8, 23.6, and 46.7 µM). Cell viability was assessed every 24 h for 5 days. **(F-I)** Effects of PNSC928 on gene expression levels. The RAW264.7 cells were co-treated with LPS and PNSC928 (23.6 and 47.2 µM) for a duration of 6 h. Subsequently, RNA isolation and RT-qPCR analysis were performed to quantify mRNA levels of various genes. **(F)** The mRNA levels of IL-1B, IL-6, IL-15, IL-18, TNFA, and IFNG. **(G)** The mRNA levels of CDH1, CDKN1A, CDKN2A, CHRD, PAX6, and BAX. **(H)** The mRNA levels of NOD2, CCL2, CXCL10, CXCL12, MMP1, and STAT1 mRNA levels, **(I)** The mRNA levels of MIP2, CCR5, GM-CSF, COX2, and iNOS. *n* = 3 for each experiment. ns: no significant difference. **P < 0.01; ***P < 0.001

To assess the cytotoxicity of PNSC928, several normal cell lines including MPAEpiC, RAW264.7, FL83B, TKPTS, and HL-1 were cultured. The cell viability assay indicated that PNSC928, at lower concentrations (≤ 23.6 µM), did not inhibit cell growth across all tested cell lines (Fig. 5A and E). A slight inhibition of cell growth was observed at a concentration of 47.2 µM of PNSC928 in the medium, implying that high concentrations of PNSC928 might exhibit weak cytotoxicity (Fig. 5A and E).

Subsequently, we explored the inhibitory impacts of PNSC928 on proinflammatory cytokine genes and other downstream target genes of CtBP2, p300, and NF-κB. In RAW264.7 cells treated with LPS, PNSC928 supplementation demonstrated a dose-dependent reversal of LPS-induced IL-1B, IL-6, IL-15, IL-18, TNFA, and IFNG expressions (Fig. 5F). Intriguingly, PNSC928 treatments did not alter the expression levels of CDH1, CDKN1A, CDKN2A, CHRD, PAX6, BAX, NOD2, CCL2, CXCL10, CXCL12, MMP1, STAT1, MIP2, CCR5, GM-CSF, VEGF, COX2, and iNOS (Fig. 5G and I). This suggests that at its IC_50_ concentration, PNSC928 doesn’t display marked cytotoxicity. Moreover, it appears to have greater specificity compared to inhibitors for CtBP2 (MTOB and NSC95397), p300 (C646 and A-485), and NF-κB (TPCA1 and BOT64).

#### Administration of PNSC928 in ARDS mice markedly reduced inflammation and enhanced recovery outcomes

Further investigations were conducted to assess the in vivo cytotoxicity of PNSC928. Mice administered with varying doses (0, 5, 10, and 20 mg/kg) of PNSC928 at two-day intervals for a duration of 6 days exhibited no discernible alterations in activities and levels of ALT, AST, ALP, BUN, and creatinine (Figures S10A-S10E), suggesting that PNSC928 may have no impact on liver and kidney functions. PNSC928 also did not affect mouse body weights (Figure S10F) or behavioral activities, such as feeding, balance ability, and sleeping patterns (data not shown). Histological examinations revealed no conspicuous damage to the lung, heart, liver, and kidney, suggesting a negligible toxicological profile of PNSC928 in mice (Figure S10G).

**Fig. 6:**
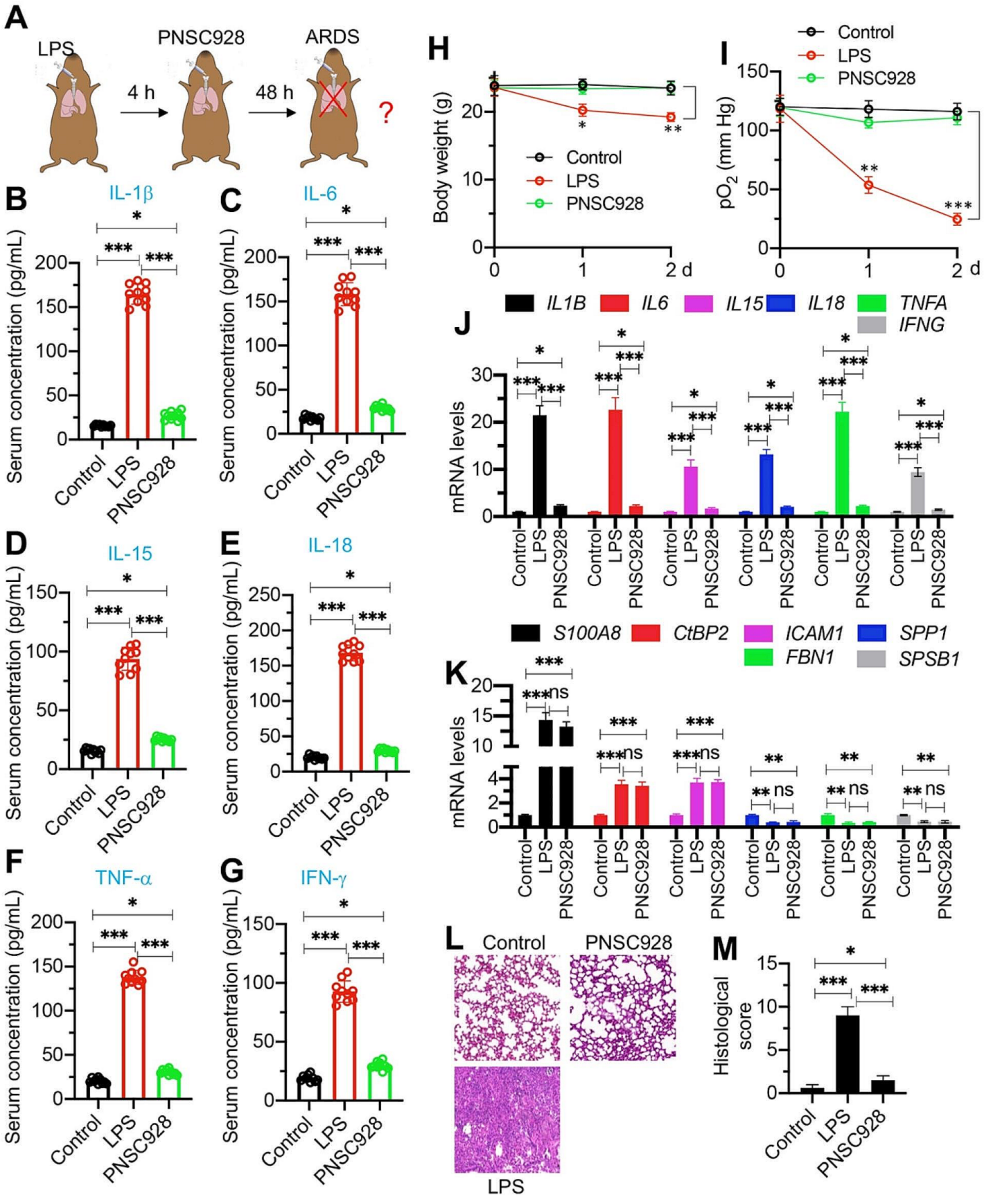
Administration of PNSC928 significantly improve the inflammatory outcomes of ARDS mice. **(A)** A schematic representation illustrating PNSC928 administration. **(B-G)** Serum concentrations of proinflammatory cytokines by ELISA assays. **(B)** IL-1β, **(C)** IL-6, **(D)** IL-15, **(E)** IL-18, **(F)** TNF-α, and **(G)** IFN-γ. **(H**) Body weights of mice measured at 0, 1, and 2 days. **(I)** pO_2_ levels in mice measured at 0, 1, and 2 days. **(J)** Effects of PNSC928 on the expression levels of IL-1B, IL-6, IL-15, IL-18, TNFA, and IFNG. **(K)** Effects of PNSC928 on the expression levels of S100A8, CtBP2, ICAM1, SPP1, FBN1, and SPSB1. **(L)** Representative H&E staining images of lung from control, ARDS, PNSC928 groups of mice. Bars = 100 μm. **(M)** Quantification of histological scores. Images in (L) were quantified. *n* = 3 for each experiment. ns: no significant difference. *P < 0.05; **P < 0.01; ***P < 0.001

Subsequently, to scrutinize the in vivo efficacy of PNSC928 in ameliorating ARDS outcomes, this compound was administered to mice challenged with LPS (four hours post-LPS administration) (Fig. 6A). ELISA assays underscored that PNSC928 significantly abated the serum and BALF concentrations of pro-inflammatory cytokines, albeit their levels were observed to be elevated (1.3–1.5 fold) in comparison to control mice (Fig. 6B and G and S11A-S11F). Following PNSC928 administration, the decrease in body weights and respiratory distress caused by LPS were alleviated (Fig. 6H and I). Importantly, the administration of PNSC928 resulted in a significant reduction in total BALF protein levels, MPO activity, and lung wet-to-dry weight ratio, while enhancing the survival rate of ARDS mice. (Figures SS11G-S11J).

Moreover, we assessed PNSC928’s impact on the expression of IL-1B, IL-6, IL-15, IL-18, TNFA, IFNG, S100A8, ICAM1, SPP1, FBN1, and SPSB1, all identified as dysregulated in ARDS lung tissues. RT-qPCR confirmed that PNSC928 significantly reduced the expressions of IL-1B, IL-6, IL-15, IL-18, TNFA, and IFNG without notably altering S100A8, ICAM1, SPP1, FBN1, and SPSB1 (Fig. 6J and K). This indicates PNSC928’s selective efficiency in regulating pro-inflammatory cytokine gene expressions without affecting other genes. Histological examinations revealed that PNSC928 recovered lung damage in LPS-challenged mice (Fig. 6L and M). These comprehensive in vivo studies underline PNSC928’s potential in reducing inflammation and enhancing ARDS outcomes without causing noticeable toxicity or unwanted gene expression changes, highlighting its therapeutic promise.

## Discussion

**Fig. 7:**
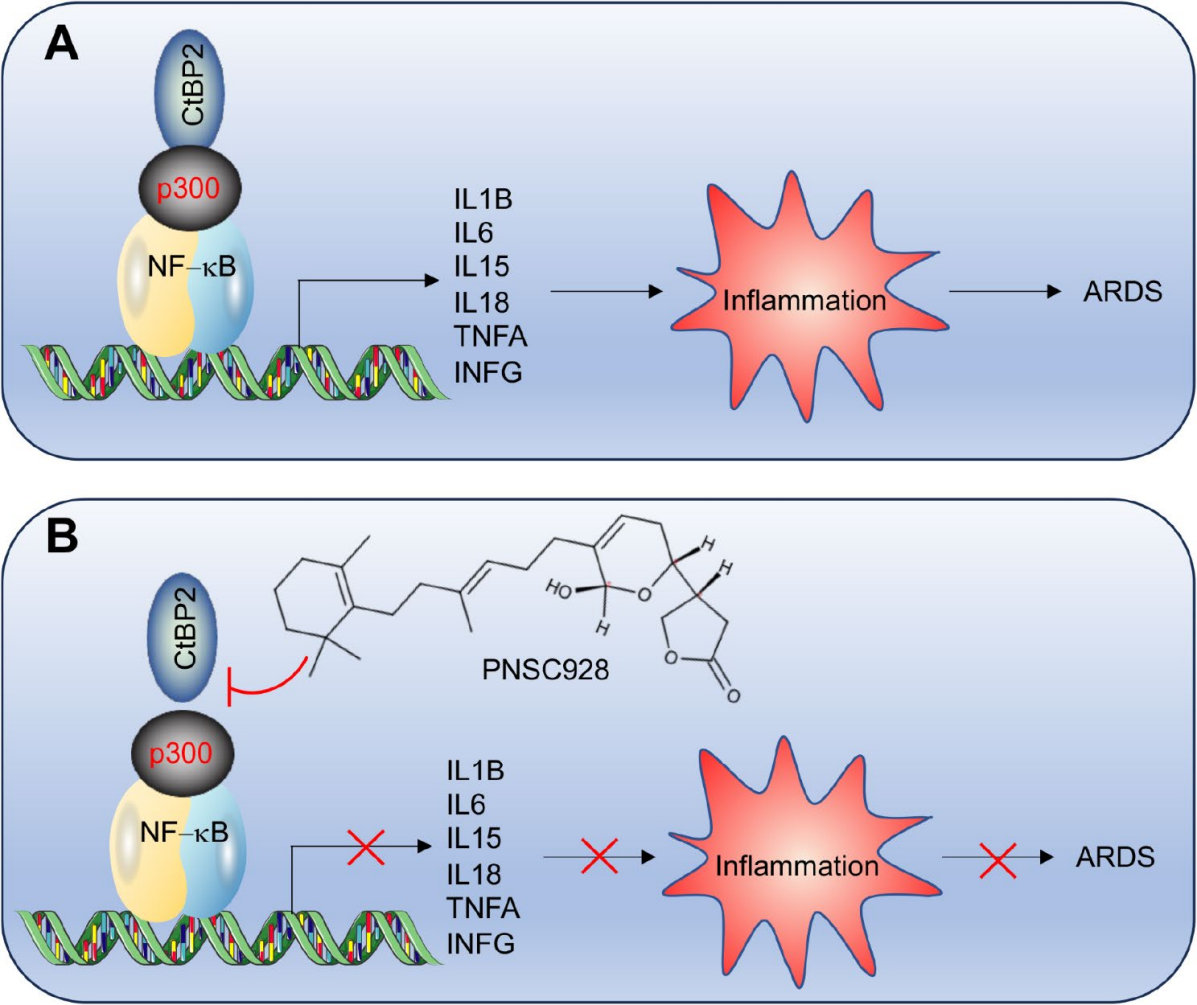
A schematic model of targeting CtBP2-p300 by PNSC928 to suppress the expression of proinflammatory cytokine genes and improve ARDS outcomes. **(A)** This schematic model illustrates the role of the CtBP2-p300-NF-κB complex in the activation of proinflammatory cytokine genes. CtBP2 forms a transcriptional complex with p300 and NF-κB subunits, leading to the activation of proinflammatory cytokine genes (IL-1B, IL-6, IL-15, IL-18, TNFA, and IFNG). The induction of these proinflammatory cytokines promotes the inflammatory response, contributing to the pathogenesis of ARDS. **(B)** This schematic model demonstrates the mechanism of action of PNSC928 in targeting the CtBP2-p300 complex. PNSC928 specifically disrupts the interaction between CtBP2 and p300, effectively suppressing the expression of proinflammatory cytokine genes. This intervention ultimately leads to improved outcomes in ARDS by mitigating the inflammatory response

The role of inflammation as a primary contributor to ARDS is well established, but critical aspects of this process remain enigmatic [1, 2]. Primarily, the identification of specific proinflammatory and anti-inflammatory cytokines integral to the inflammatory response in ARDS is yet to be fully elucidated [1, 2]. Secondly, the absence of robust therapeutic agents capable of selectively mitigating inflammation in ARDS without inducing adverse side effects presents a substantial challenge [1, 2]. A profound understanding of the molecular mechanisms underpinning this condition is paramount for the formulation of targeted therapeutic interventions [1, 2]. In this study, we employed RNA-Seq to explore DEGs in ARDS-afflicted mice, discovering predominant inductions of IL-1β, IL-6, IL-15, IL-18, TNFA, and IFNG following LPS challenges. Notably, we ascertained that these proinflammatory genes are meticulously regulated by the CtBP2-p300-NF-κB complex (Fig. 7A). A pivotal advancement of our work was the high-throughput identification of the compound PNSC928, which demonstrably disrupted the CtBP2-p300^BRD^ interaction, offering an intriguing perspective for further explorations. Remarkably, PNSC928 manifested negligible cytotoxicity at lower concentrations and only mild cytotoxicity at elevated concentrations. The in vitro and in vivo administration of PNSC928 presented notable specificity in inhibiting the expressions of IL-1B, IL-6, IL-15, IL-18, TNFA, and IFNG, without significantly altering the expressions of other target genes of CtBP2, p300, and NF-κB. Consequently, it rendered substantial improvement in the outcomes of ARDS in mice (Fig. 7B), indicating its potential as a novel therapeutic candidate for the management of ARDS.

The therapeutic management of ARDS faces substantial challenges, primarily due to the complexities involved in mitigating inflammatory responses [3–6]. While inflammation is a natural protective mechanism, in ARDS, an exaggerated inflammatory response leads to pulmonary damage, thus necessitating controlled inhibition of inflammation [1–4]. However, striking a balance between adequate suppression of inflammation and averting impairment of the body’s normal immune response is intricate and fraught with difficulties [1–4]. Several anti-inflammatory agents (e.g., bosentan and dexamethasone) have been explored for ARDS therapy, but they often exhibit a lack of specificity, affecting not only the pathways leading to inflammation but also other crucial physiological pathways, potentially leading to unintended consequences and side effects [3, 4, 30]. Additionally, the heterogeneity in ARDS etiologies and presentations further complicate the development of universally effective anti-inflammatory therapies, necessitating a multifaceted approach to understand and address the specific underlying inflammatory processes and pathways [3, 4, 30]. The pursuit for selective and targeted anti-inflammatory agents that can effectively mitigate the unregulated inflammatory cascades in ARDS, without compromising other physiological functions, remains a pivotal challenge in advancing ARDS therapeutics [3, 4, 30]. Our insights are anchored in comprehensive RNA-seq analyses on ARDS lungs and LPS-treated CtBP2^KD^/p300^KD^/p65^KD^ cells, which emphasized the pivotal role of IL-1β, IL-6, IL-8, IL-15, TNF-α, and IFN-γ in LPS-challenged ARDS and the important role of the CtBP2-p300-NF-κB complex in modulating proinflammatory genes, with minor influence on other CtBP2, p300, and NF-κB target genes. This precise modulation underscores the specificity of this complex in the inflammatory cascade of ARDS, a finding that holds significant therapeutic promise.

While several inhibitors targeting CtBP2, p300, and NF-κB have been reported, the specificity and cytotoxicity of these inhibitors largely remain elusive, particularly within lung cells/tissues and the immune system [15, 24, 25]. We systematically evaluated the cytotoxicity and specificity of varying doses of CtBP2, p300, and NF-κB inhibitors, discovering that they manifested moderate to high levels of cytotoxicity across lung, kidney, liver, and heart cells and tissues. Despite their proficiency in significantly attenuating the expression levels of proinflammatory cytokine genes, these inhibitors also demonstrated the propensity to modulate the expression of other CtBP2, p300, and NF-κB target genes. This suggests potential unsuitability as therapeutic agents for ARDS treatment, due to possible unintended molecular consequences. Our experimental data illustrated that PNSC928 is not simply an inhibitor but acts strategically to impede the assembly of the CtBP2-p300-NF-κB complex. This impeding action resulted in a pronounced reduction in the expression of proinflammatory cytokine genes within LPS-treated macrophages and lung epithelial cells. Importantly, our experiments demonstrated that PNSC928 is not merely an inhibitor; it actively obstructs the assembly of the CtBP2-p300-NF-κB complex. This obstruction precipitated a marked reduction in the expression of proinflammatory cytokine genes in LPS-treated macrophages and lung epithelial cells. Remarkably, this potent inhibition was manifested without inducing any discernible cytotoxic effects across various normal cell types, emphasizing its safety profile. Furthermore, we investigated the effects of PNSC928 on key molecules within the NF-κB signaling pathway, including the phosphorylation levels of IKKα, protein levels of IκBα, and nuclear translocation of p65 and p50. Our findings revealed that treatment with PNSC928 did not affect the phosphorylation, protein expression, or nuclear translocation of these molecules induced by LPS. These results suggest that PNSC928 inhibits the expression of proinflammatory cytokine genes through the suppression of the CtBP2-p300 interaction, rather than affecting other molecules in the NF-κB signaling pathway.

In vivo applications of PNSC928 displayed profound impacts, particularly in mitigating CtBP2-driven proinflammatory gene expressions in ARDS mice models. This mitigation translated into significant alleviations in inflammation and subsequent lung injuries, optimizing the overall prognosis of ARDS. The specificity of PNSC928 in targeting proinflammatory genes, without substantive alterations to other CtBP2, p300, and NF-κB target genes, is indicative of its therapeutic precision and reduced likelihood of off-target effects. It’s crucial to underscore that while inhibiting CtBP2, p300, or NF-κB demonstrated improved inflammatory symptoms in ARDS mice models, it also unveiled the onset of side effects attributed to the alteration in the expressions of other target genes, emphasizing the need for precise therapeutic interventions.

Although our in vitro and in vivo results suggest that PNSC928 is a potential therapeutic molecule for ARDS, further comprehensive research is warranted to substantiate this conclusion. A comprehensive evaluation of both in vitro and in vivo toxicity is essential for the clinical application of PNSC928. This includes broader assessments of its effects on cell proliferation and apoptosis across additional normal cell lines, as well as genome-wide analyses of its impact on gene and protein expression in PNSC928-treated cells. Additionally, toxicity assessments at the animal level should encompass analyses of organ-specific effects in mice following treatment with a wider range of doses and longer durations.

## Conclusion

This study unravels PNSC928 as a promising candidate in ARDS therapeutic research, by not only pinpointing its potential to disrupt the CtBP2-p300 interaction but also by highlighting its role as a powerful, selective, and safe inhibitor of proinflammatory gene expressions. The prospects of utilizing PNSC928 in clinical settings offer a promising avenue to attenuate the debilitating impacts of inflammation in ARDS, paving the way for more nuanced and targeted approaches in managing and possibly reversing the intricate dynamics of this severe pulmonary condition. Future studies and clinical trials will be instrumental in validating these findings and exploring the full therapeutic potential and applicability of PNSC928 in ARDS and possibly other inflammatory conditions.

### Electronic supplementary material

Below is the link to the electronic supplementary material.

Supplementary Material 1

## Data Availability

No datasets were generated or analysed during the current study.
