# Supplementary material for: PNSC928, a plant-derived compound, specifically disrupts CtBP2-p300 interaction and reduces inflammation in mice with acute respiratory distress syndrome
Source: Biol Direct. 2024 Jun 21;19:48. doi: 10.1186/s13062-024-00491-0 (PMC11191317; doi:10.1186/s13062-024-00491-0)
Supplement: Supplementary file 1 — Supplementary Material 1 [file 13062_2024_491_MOESM1_ESM.docx]

**
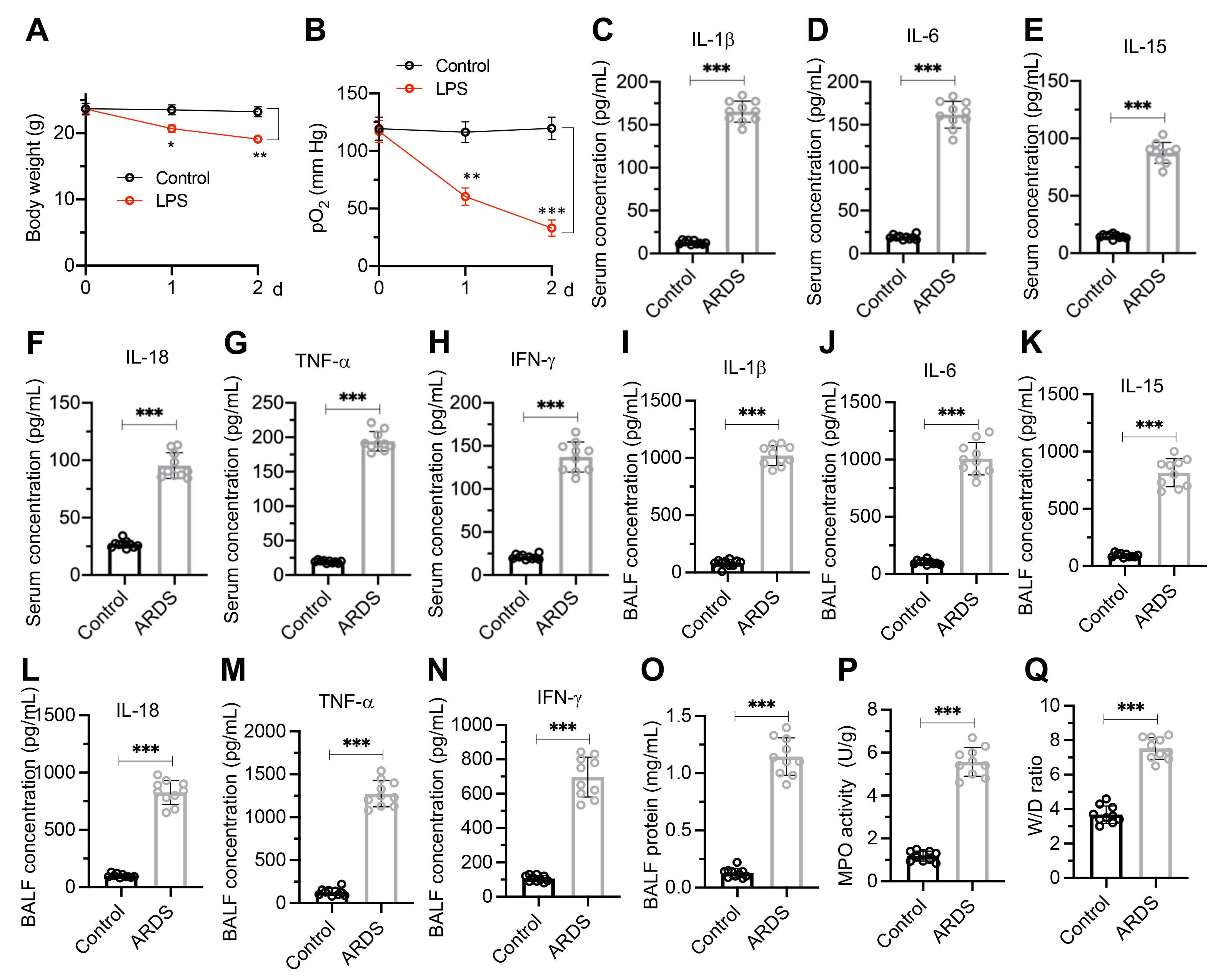
**

**Figure S1. Proinflammatory cytokine levels, body weights, and pO_2_ levels in ARDS mice**

**(A-F)** Serum concentrations of proinflammatory cytokines, including IL-1β **(A)**, IL-6 **(B)**, IL-15 **(C)**, IL-18 **(D)**, TNF-α **(E)**, INF-γ **(F),** in both Control and ARDS groups of mice (n=10 for each group). **(G-L)** BALF concentrations of proinflammatory cytokines, including IL-1β **(G)**, IL-6 **(H)**, IL-15 **(I)**, IL-18 **(J)**, TNF-α **(K)**, INF-γ **(L),** in both Control and ARDS groups of mice (n=10 for each group). **(M**) Total protein level in BALF (n=10 for both control and ARDS groups of mice). **(N)** MPO activity in lung tissues (n=10 for both control and ARDS groups of mice). **(O**) Lung wet/dry weight ratio (n=10 for both control and ARDS groups of mice). **(P)** Body weights of mice measured at 0, 1, and 2 days. **(Q)** pO_2_ levels in mice measured at 0, 1, and 2 days (n=10 for both control and ARDS groups of mice). **P*<0.05; ***P*<0.01; ****P*<0.001.

**
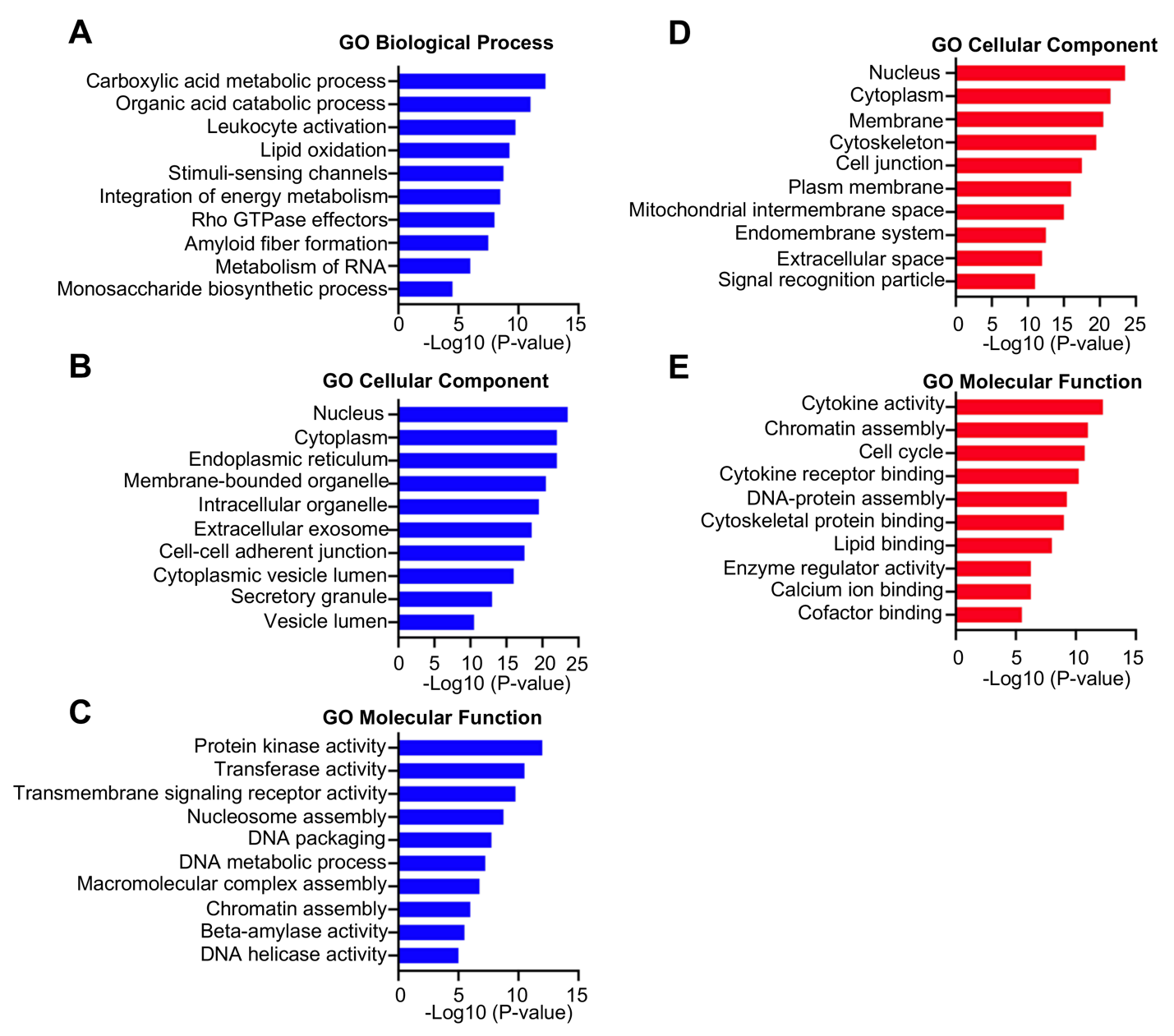
**

**Figure S2. Gene ontology enrichment analysis of differentially expressed genes in ARDS mice**

GO biological process, cellular component, and molecular function enrichment analyses of downregulated **(A-C)** and upregulated **(D-E)** genes in ARDS mice were conducted using MetaCore bioinformatics software.

**
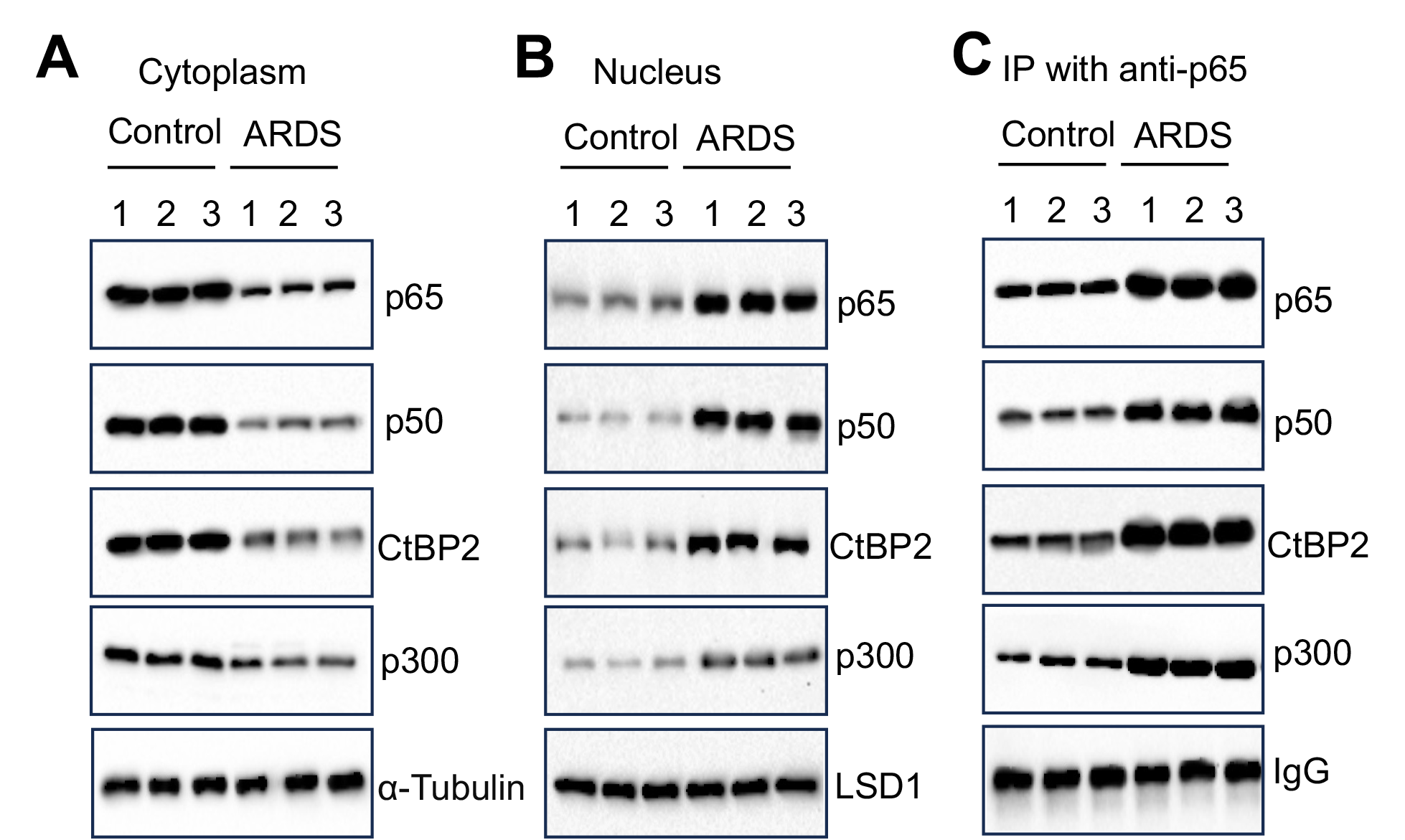
**

**Figure S3. Protein levels of CtBP2-p300-NF-κB complex members in cytoplasm and nucleus**

Cytoplasmic and nuclear proteins were isolated from lung tissues of control and ARDS mice (n=3 for each group). Immunoblots were performed to assess protein levels of p65, p50, CtBP2, and p300 in the cytoplasmic fraction **(A)** and nuclear fraction **(B)**. α-tubulin served as a loading control for cytoplasmic proteins, while LSD1 (lysine demethylase 1A) served as a loading control for nuclear proteins. **(C)** Immunoprecipitation results: Nuclear proteins used in (B) were immunoprecipitated using anti-p65-binding protein G agarose, and the outputs were used to detect protein levels of p65, p50, CtBP2, and p300.


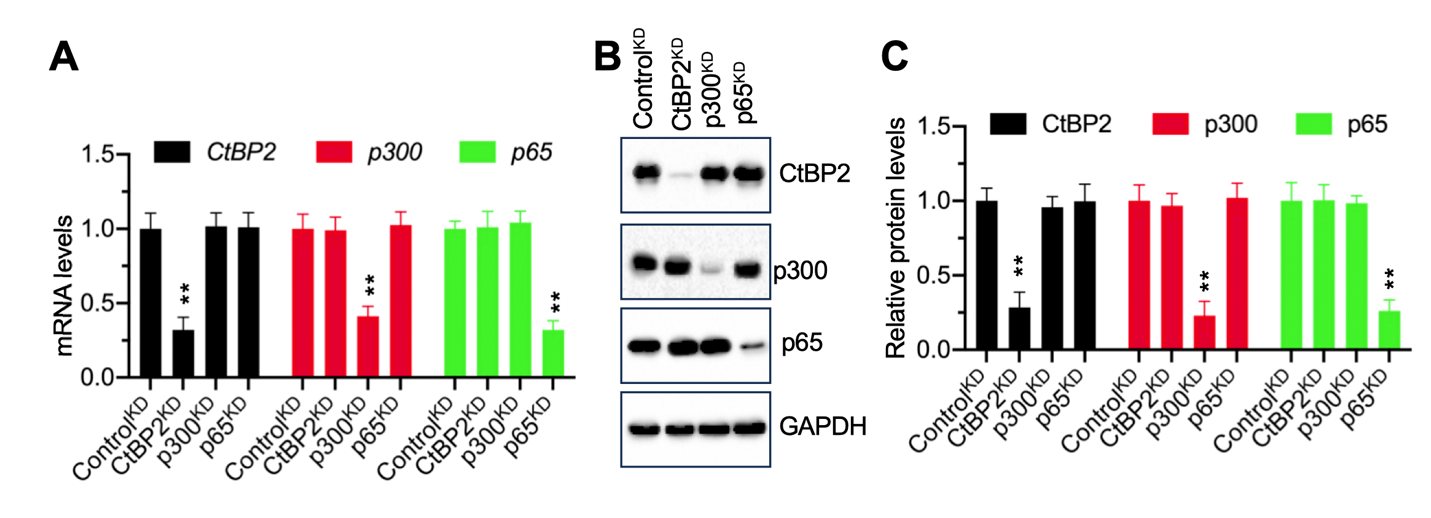


**Figure S4. mRNA and protein levels of CtBP2-p300-NF-κB complex members in corresponding knockdown cell lines**

**(A)** mRNA levels of CtBP2, p300, and p65 were determined using RT-qPCR analysis with RNA samples from Control^KD^, CtBP2^KD^, p300^KD^, and p65^KD^ cells (n=3). **(B and C)** Protein levels of CtBP2, p300, and p65 were assessed by immunoblots using protein samples from Control^KD^, CtBP2^KD^, p300^KD^, and p65^KD^ cells (n=3).

**
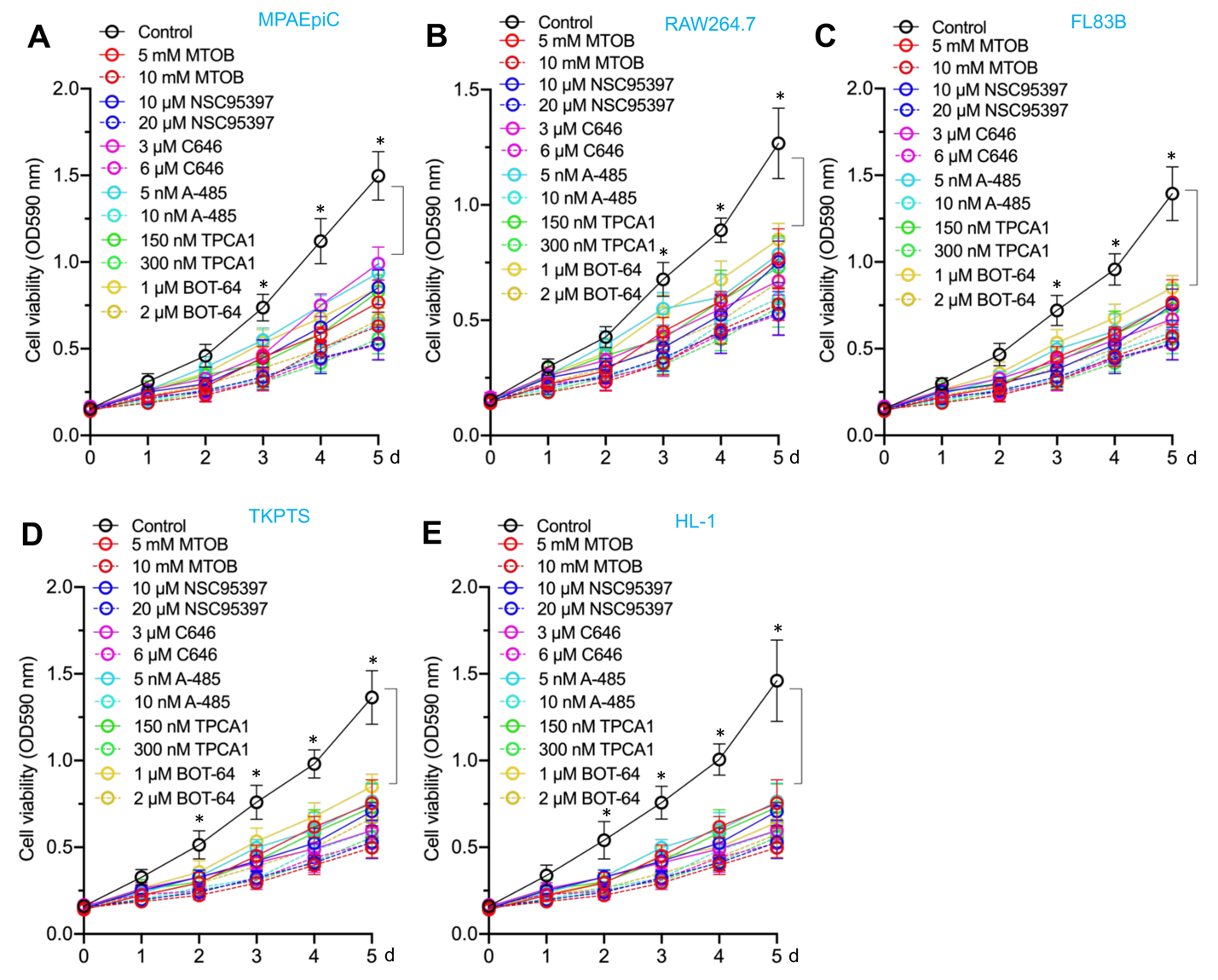
**

**Figure S5. Inhibition of CtBP2, p300, and NF-κB significantly reduces cell viability**

Five cell lines (MPAEpiC, RAW264.7, FL83B, TKPTS, and HL-1) were treated with MTOB (5 and 10 mM), NSC95397 (10 and 20 μM), C646 (3 and 6 μM), A-485 (5 and 10 nM), TCPA1 (150 and 300 nM), and BOT-64 (1 and 2 μM). Cell viability was assessed every 24 hours for 5 days. n=3 for all experiments. **P*<0.05.


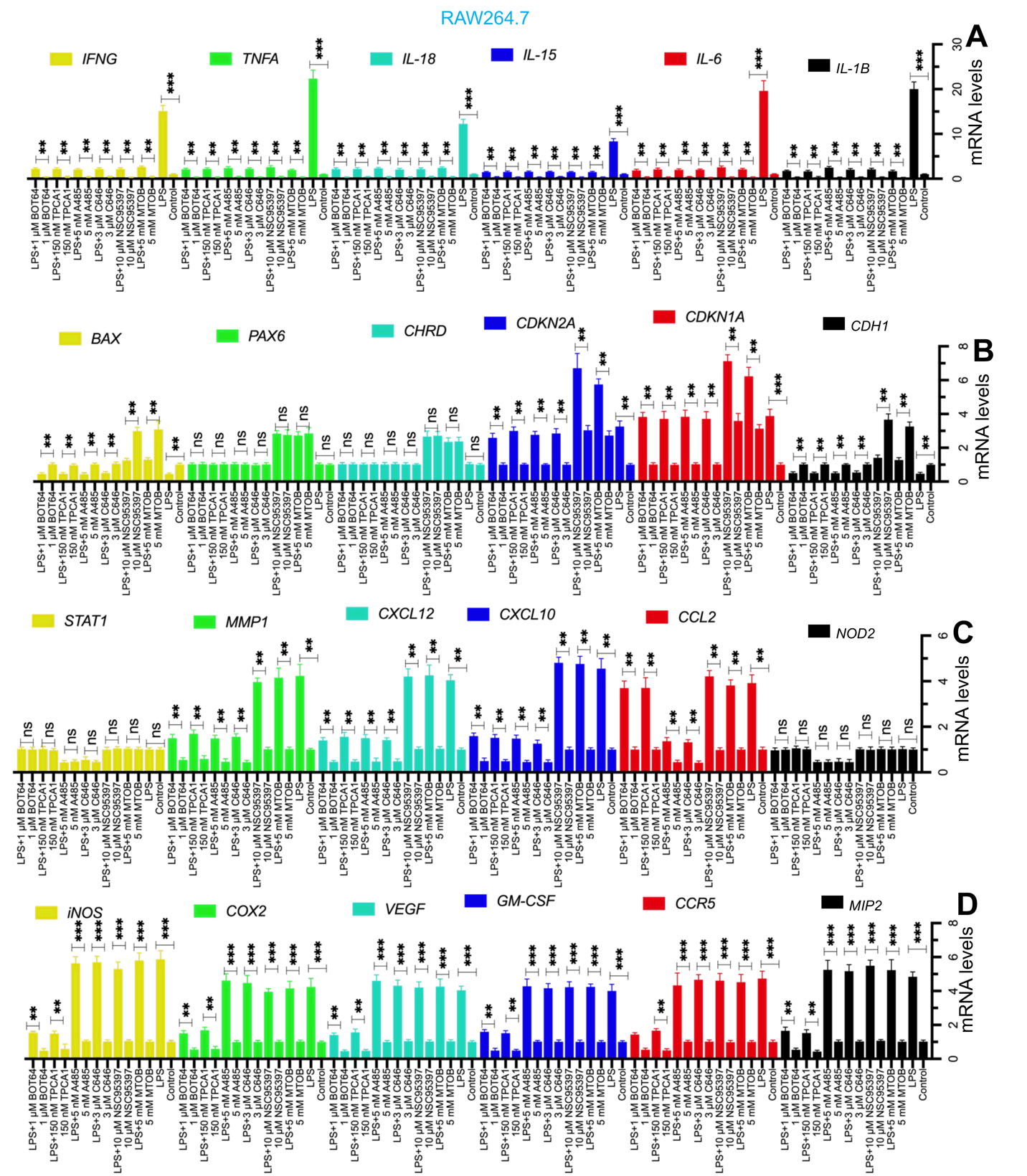


**Figure S6. Effects of CtBP2, p300, and NF-κB inhibitors on the expression levels of their target genes in RAW264.7 cells**

The RAW264.7 cells were co-treated with LPS and the following compounds: 5 mM MTOB, 10 μM NSC95397, 3 μM C646, 5 nM A-485, 150 nM TPCA1, or 1 μM BOT64 for a duration of 6 hours. Subsequently, RNA isolation and RT-qPCR analysis were performed to quantify mRNA levels of various genes. **(A)** The mRNA levels of IL-1B, IL-6, IL-15, IL-18, TNFA, and IFNG. **(B)** The mRNA levels of CDH1, CDKN1A, CDKN2A, CHRD, PAX6, and BAX. **(C)** The mRNA levels of NOD2, CCL2, CXCL10, CXCL12, MMP1, and STAT1 mRNA levels, **(D)** The mRNA levels of MIP2, CCR5, GM-CSF, COX2, and iNOS. ns: no significant difference. n=3 for all experiments. ***P*<0.01; ****P*<0.001.


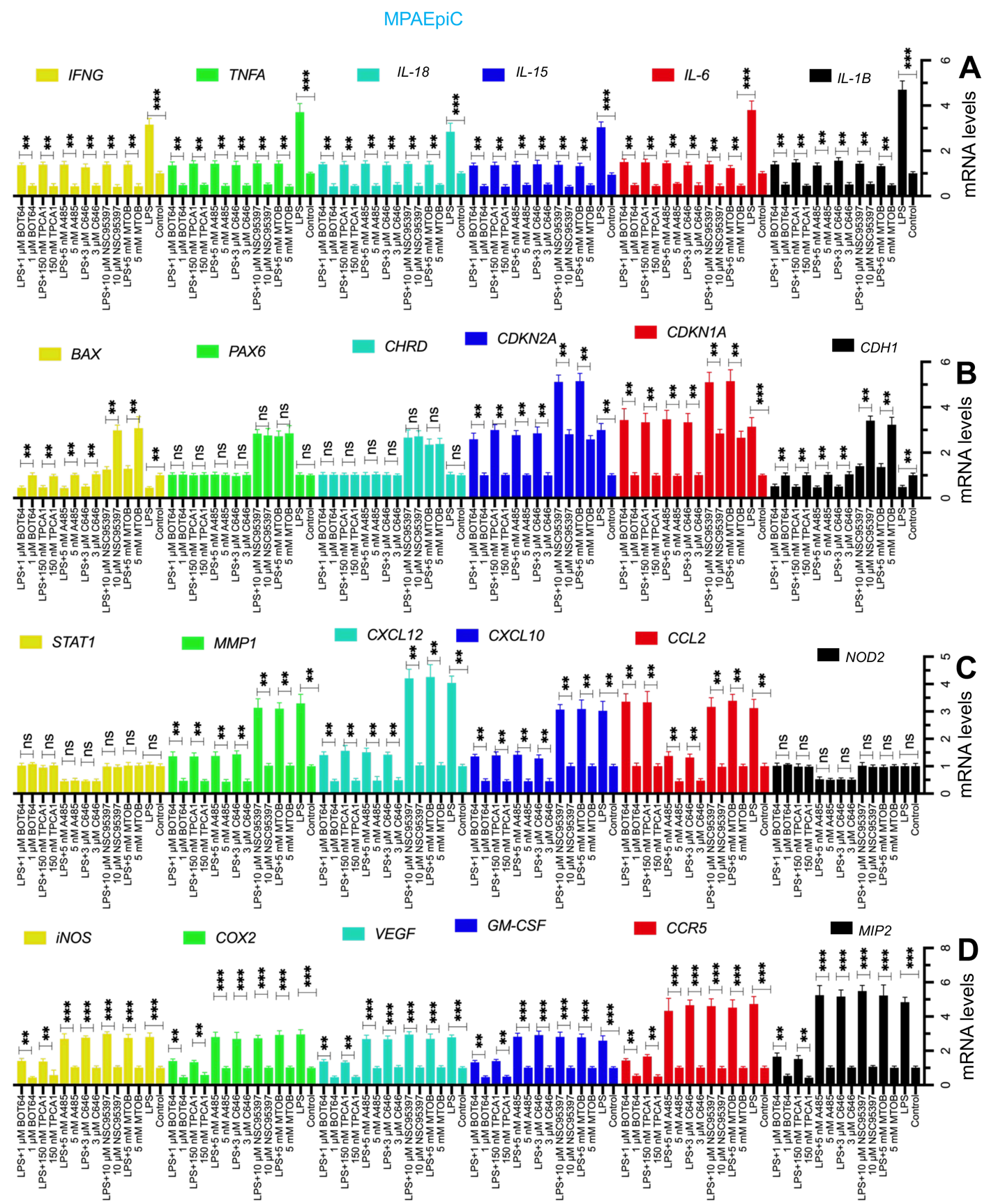


**Figure S7. Effects of CtBP2, p300, and NF-κB inhibitors on the expression levels of their target genes in MPAEpiC cells**

The MPAEpiC cells were co-treated with LPS and the following compounds: 5 mM MTOB, 10 μM NSC95397, 3 μM C646, 5 nM A-485, 150 nM TPCA1, or 1 μM BOT64 for a duration of 6 hours. Subsequently, RNA isolation and RT-qPCR analysis were performed to quantify mRNA levels of various genes. **(A)** The mRNA levels of IL-1B, IL-6, IL-15, IL-18, TNFA, and IFNG. **(B)** The mRNA levels of CDH1, CDKN1A, CDKN2A, CHRD, PAX6, and BAX. **(C)** The mRNA levels of NOD2, CCL2, CXCL10, CXCL12, MMP1, and STAT1 mRNA levels, **(D)** The mRNA levels of MIP2, CCR5, GM-CSF, COX2, and iNOS. ns: no significant difference. n=3 for all experiments. ***P*<0.01; ****P*<0.001.

**
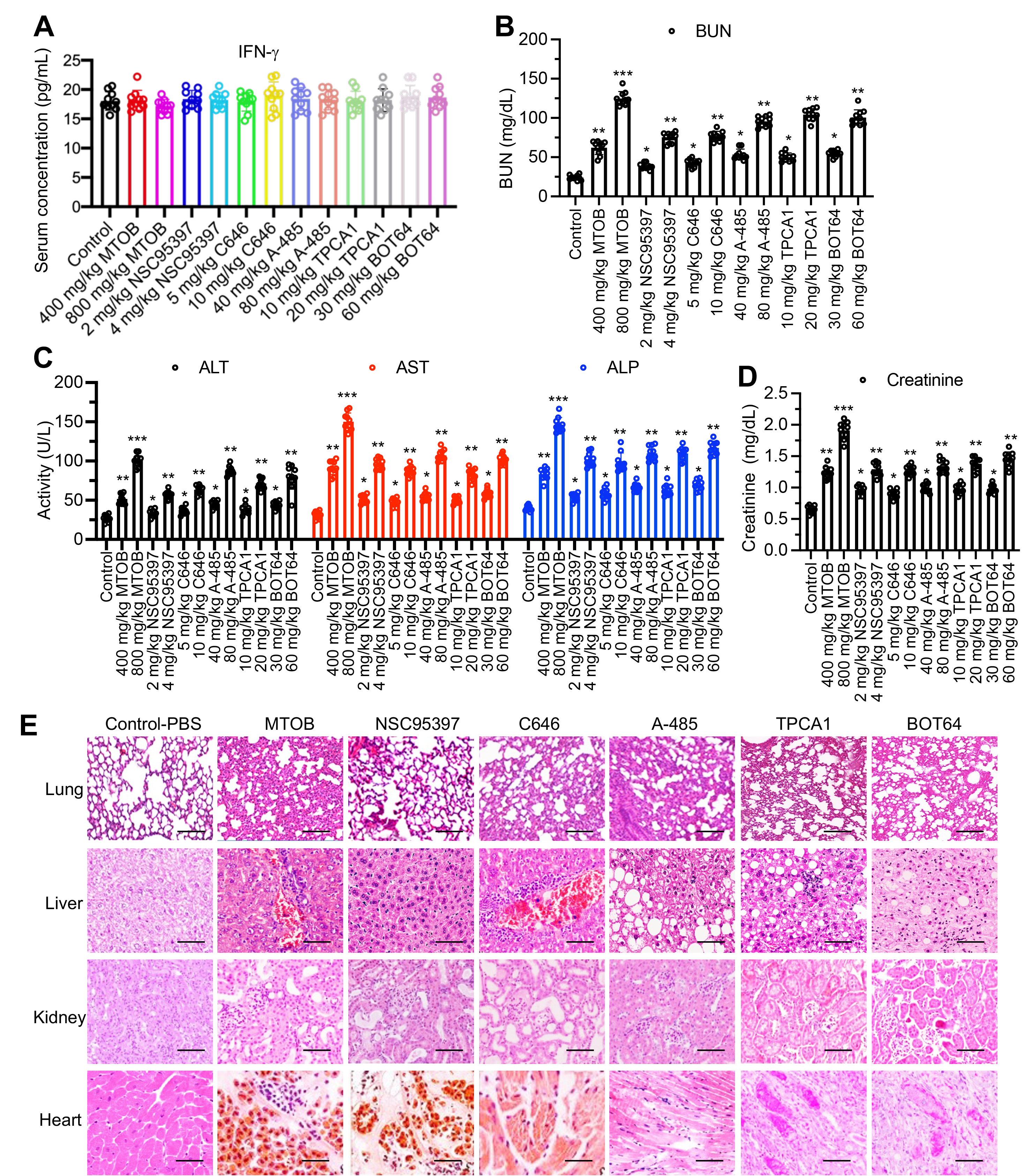
**

**Figure S8. Inhibitors of** **CtBP2, p300, and NF-κB caused damages to mouse lung, liver, kidney, and heart**

C57BL/6 mice were administrated with MTOB (400 and 800 mg/kg), NSC95397 (2 and 4 mg/kg), C646 (5 and 10 mg/kg), A-485 (40 and 80 mg/kg), TPCA1 (10 and 20 mg/kg), and BOT64 (30 and 60 mg/kg) for a duration of 6 days (n=10 for each group). **(A)** Serum concentration of IFN-γ in different groups of mice. **(B)** Serum BUN concentration. **(C)** Activities of ALT, AST, and ALP in serum. **(D)** Serum creatinine concentration. **(E)** Representative H&E staining images of lung, liver, kidney, and heart from the following groups of mice: Control, MTOB (800 mg/kg), NSC95397 (4 mg/kg), C646 (10 mg/kg), A-485 (80 mg/kg), TPCA1 (20 mg/kg), and BOT64 (60 mg/kg). Bars=100 μm. For the significant difference analysis in B-D, all drug-treated groups were compared with the Control group of mice. **P*<0.05; ***P*<0.01; ****P*<0.001.

**
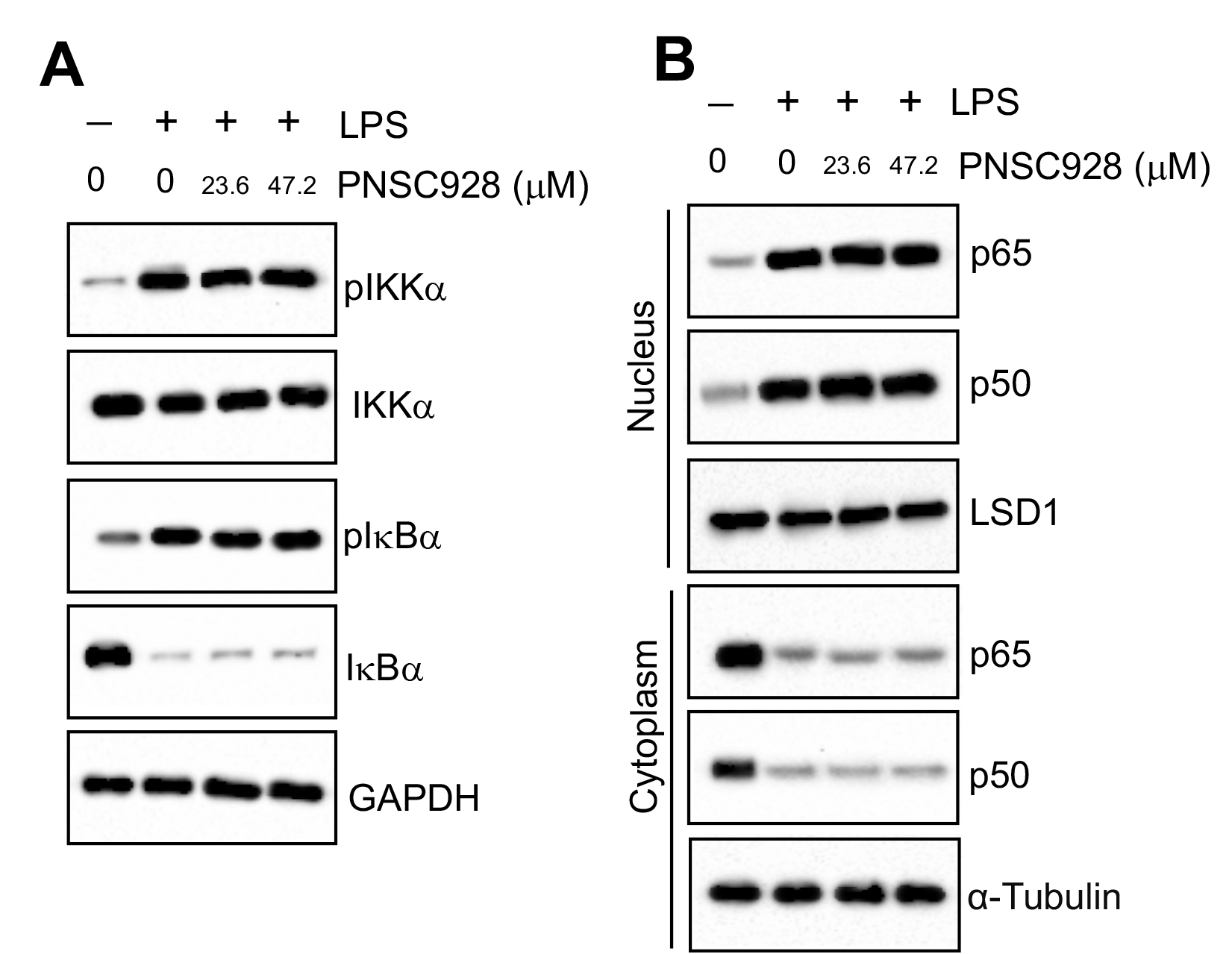
**

**Figure S9. Effect of PNSC928 on the upstream signaling molecules of NF-κB**

The MPAEpiC cells were co-treated with LPS (200 ng/mL) and different concentrations (0, 23.6, and 47.2 μM) of PNSC928 for 6 hours, followed by PBS washing three times and protein extraction. **(A)** Protein levels of pIKKα, IKKα, pIκBα, IκBα, and GAPDH (Glyceraldehyde 3-phosphate dehydrogenase, loading control). **(B)** The protein levels of p65 and p50 in cytoplasmic and nuclear fractions. LSD1 was used as a nuclear loading control, and α-tubulin was used as a cytoplasmic loading control. n=3 for each experiment.

**
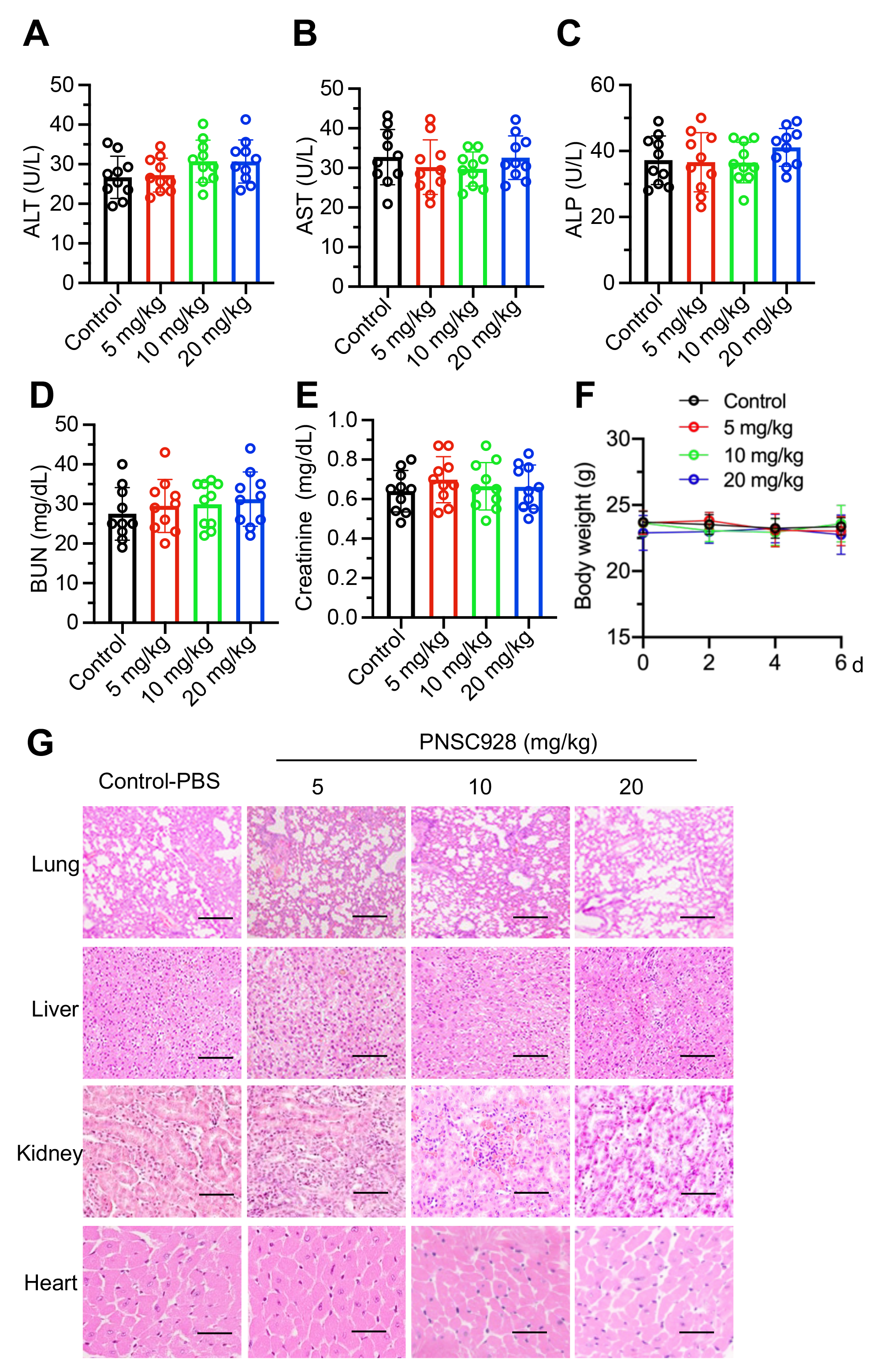
**

**Figure S10. PNSC928 could not change mouse body weights and cause damages to mouse lung, liver, kidney, and heart**

C57BL/6 mice were administrated with PNSC928 (5, 10, and 200 mg/kg, n=10 for each dose) for a duration of 6 days. **(A)** Serum ALT activity. **(B)** Serum AST activity. **(C)** Serum ALP activity. **(D)** Serum BUN concentration. **(E)** Serum creatinine concentration. **(F)** Body weights were recorded every two days. **(G)** Representative H&E staining images of lung, liver, kidney, and heart. Bars=100 μm.

**
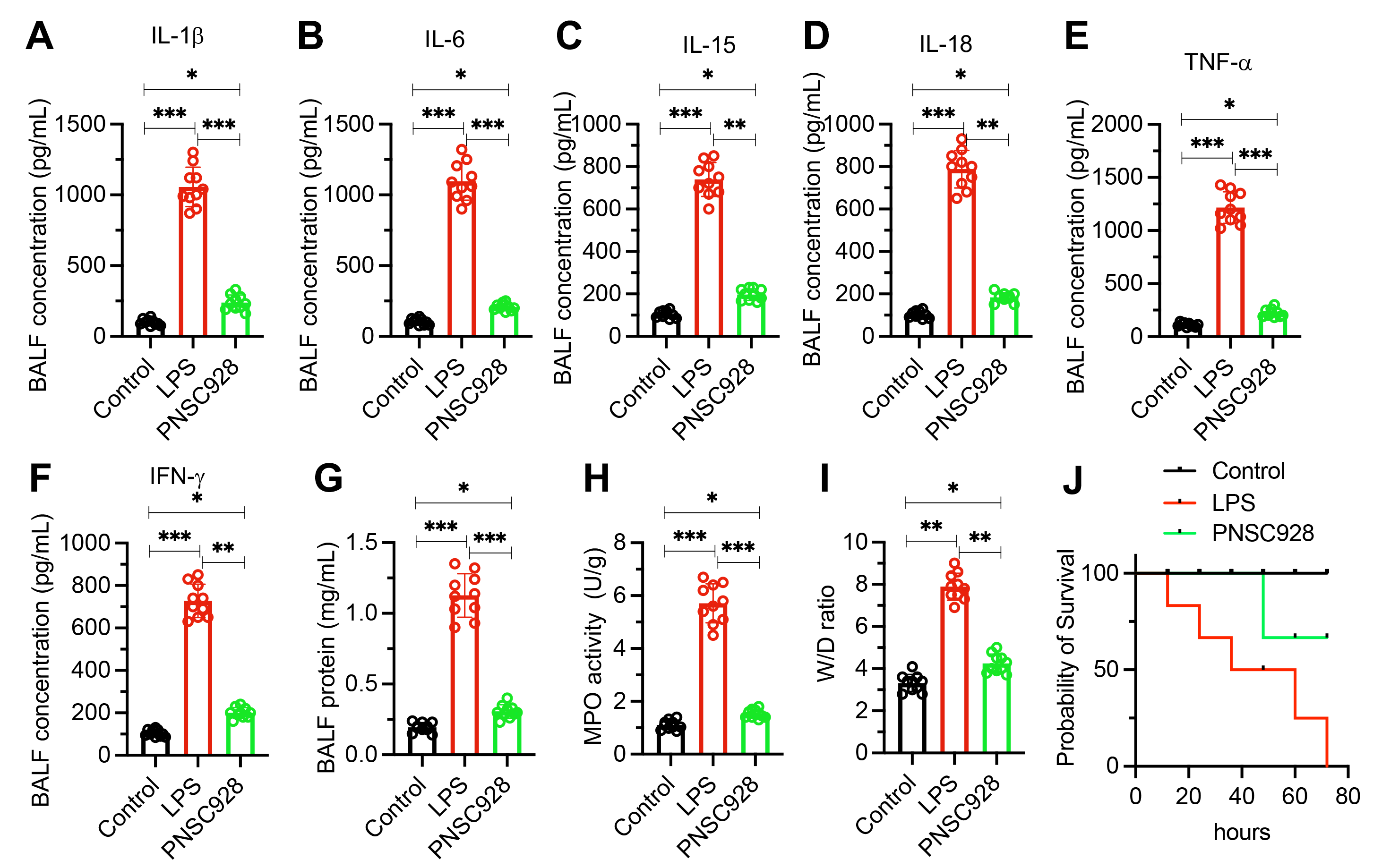
**

**Figure S11. Effects of PNSC928 on BALF concentrations of proinflammatory cytokines, MPO activity, wet/dry lung weight ratio and survival rate**

C57BL/6 mice were randomly divided into three groups and administered sterile saline, 25 μg LPS, and 25 μg LPS+20 mg/kg PNSC928, respectively (n=10 for each group). **(A-F)** BALF concentrations of proinflammatory cytokines by ELISA assays. **(A)** IL-1β, **(B)** IL-6, **(C)** IL-15, **(D)** IL-18, **(E)** TNF-α, and **(F)** IFN-γ. **(G)** Total protein levels in BALF. **(H)** MPO activity in lung tissues. **(I)** Lung wet/dry weight ratio. **(J)** Survival rate of different groups of mice. **P*<0.05; ***P*<0.01; ****P*<0.001.

**Table S1. Information of short hairpin RNAs (shRNAs) specifically targeting genes**

| **Gene** | **TRC Clone ID** | **Sources** |
| --- | --- | --- |
| CtBP2 | TRCN0000109336 | Sigma-Aldrich, China |
|  | TRCN0000109337 |  |
| p300 | TRCN0000071204 | Sigma-Aldrich, China |
|  | TRCN0000071206 |  |
| p65 | TRCN0000055346 | Sigma-Aldrich, China |
|  | TRCN0000055347 |  |
| p50 | TRCN0000009511 | Sigma-Aldrich, China |
|  | TRCN0000009512 |  |

**Table S2. Details of vectors used for gene overexpression and protein expression in E.coli**

| **Vectors** | **Insertion sites** | **Forward primers (5’-3’)** | **Reverse primers (5’-3’)** |
| --- | --- | --- | --- |
| pCDNA3-Flag  (Empty) | N/A | N/A | N/A |
| pCDNA3-Flag-p300^R1(1-1000 aa)^ | BamHI+XhoI | CGGGATCCATGGCCGAGAATGTGGTGGAACCC | CCGCTCGAGTTTAGCCTCCTTTGTATCCTCAGG |
| pCDNA3-Flag-p300^R2(1001-1201 aa)^ | BamHI+XhoI | CGGGATCCGGTGAGGATGTTAAAGTAGAACCTA | CCGCTCGAGACAGAAATGATACCTGTTCTGGTAAC |
| pCDNA3-Flag-p300^R3(1202-1810 aa)^ | BamHI+XhoI | CGGGATCCGAGAAGTGTTTCAATGAAATCCAA | CCGCTCGAGGATGTTGAGGCAAAATGGCACCGG |
| pCDNA3-Flag-p300^R4(1811-2412 aa)^ | BamHI+XhoI | CGGGATCCAAGCAAAAGCTCCGGCAGCAACAGC | CCGCTCGAGCTAGTGTATGTCTAGTGTACTCTG |
| pCDNA3-Myc  (Empty) | N/A | N/A | N/A |
| pCDNA3-Myc-CtBP2 | BamHI+NotI | CGGGATCCATGGCCCTTGTGGATAAGCACAAAGTC | ATAAGAATGCGGCCGCCTATTGCTCGTTGGGGTGCTCTC |
| pET28a-CtBP2 | BamHI+XhoI | CGGGATCCATGGGTGAGGATGTTAAAGTAGAACCTA | CCGCTCGAGACAGAAATGATACCTGTTCTGGTAAC |
| pGEX-6p1-p300^R2^ | BamHI+NotI | CGGGATCCATGGCCCTTGTGGATAAGCACAAAGTC | ATAAGAATGCGGCCGCCTATTGCTCGTTGGGGTGCTCTC |

**Table S3. Primer pairs specifically targeting genes for RT-qPCR analysis**

| **Genes** | **Forward primers (5’-3’)** | **Reverse primers (5’-3’)** |
| --- | --- | --- |
| IL1B | GAGCAAAGTGGAGTTTGAGTCT | GGAAGACACGGATTCCATGGT |
| IL6 | TGGCTAAGGACCAAGACCATCC | TCTGACCACAGTGAGGAATGTC |
| IL15 | CATAGCCAGCTCATCTTCAACA | TGAAGACATGAATGCCAGCCTC |
| IL18 | GCCGACTTCACTGTACAACCGCA | TGTACATGTATATTATCAGTCTG |
| TNFA | AACTCAGCGCTGAGGTCAATCTG | GTAGACAATAAAGGGGTCAGAGT |
| IFNG | GCGTCATTGAATCACACCTGAT | CCACTCGGATGAGCTCATTGAA |
| CDH1 | TGACCACGTTGGACGTCCATGT | ACAGTAGGAGCAGCAGGATCAGA |
| CDKN1A | AATGGAGACAGAGACCCCAG | TGTCCACGGGACCGAAGAGAC |
| CDKN2A | GATGATGGGCAACGTTCACGTAG | TGCAGCACCACCAGCGTGTCCA |
| CHRD | ATCTGCCAGAGACGAACAGTGAT | TGGGGAGGTCTCTACTGCGTT |
| PAX6 | GCACGGTATCAGTTGGAACAA | ATGAGCAACACAGATCCGCGAT |
| BAX | AGGATGCGTCCACCAAGAAGCTG | CATGTCAGCTGCCACCCGGAAGA |
| NOD2 | GCCCTAGCACTGATGCTGGAGAA | ATGGCACTGTTCCTGCTGAGGG |
| CCL2 | GTGTGACTCGGACTGTGAT | GTTGAATCTGGATTCACAGAGA |
| CXCL10 | CGTGGTCACATCAGCTGCTACT | TACAGTACAGAGCTAGGACAGCCA |
| CXCL12 | TAGCTGCAGAGCTGGATCCTCA | CTGGAGTGAAGATGGAAGGCTGTAT |
| MMP1 | CCAGTTACACGTGGTACACTGAAG | GGTTAGATCACCATTACATGGATGT |
| STAT1 | GTGATCACAGCCTGATGGTTCTGG | ACCGTGGTTCACACCTGCACTC |
| MIP2 | GTTGACTTCAAGAACATCCAGAGC | GGATGATTTTCTGAACCAGGG |
| CCR5 | GGAGCTTAGGAAGTAATCTGAG | CTACATTCATGTAATATGCTACC |
| GM-CSF | TGACATGCCTGTCACGTTGAATG | TAGTAGCTGGCTGTCATGTTCAAGG |
| VEGF | AGTGGTCCCAGGCTGCACCCACGA | CACAGGACGGCTTGAAGATGTACT |
| COX2 | GCTCGTTGATGAGTGGTAGCCAG | CTGAGTACCAGGCCAGCACAAA |
| NOS2 | AACACCAAGGTTGTCTGCATGGAC | AGGTGTGGTTGAGTTCTCTAAGCA |
| S100A8 | AGCAGCTGACACTTAGCCTCACAT | GAATAATTGTGGTAGACATCAATG |
| S100A9 | AGCACCTTCTCAGATGGAGCGC | CTTCATAAAGGTTGCCAACTGTGC |
| LCP1 | TGCCATTGGGTGCCACGTGGTTA | CTCCTTCTCTCAAAAGAGCAATC |
| NME2 | GAACACCTGAAGCAGCATTACATCG | AGCATCACTCGGCCCGTTTTCACC |
| SOCS1 | AGCGTATGCTTTGGCCAAACGTC | AACTAGCCTGGAAGTTTGGTCCC |
| β-actin | GGCTGTATTCCCCTCCATCG | CCAGTTGGTAACAATGCCATGT |

**Table S4. List of Antibodies Employed for Western Blot Analyses**

| **Antibodies** | **Catalog numbers** | **Dilution fold** | **Sources** |
| --- | --- | --- | --- |
| Anti-CtBP2 | #13256 | 1:1000 | Cell signaling |
| Anti-p300 | #sc-48343 | 1:2000 | Santa Cruz Biotechnology |
| Anti-p65 | #51-0500 | 1:1500 | Thermo Fisher |
| Anti-p50 | #51-3500 | 1:1500 | Thermo Fisher |
| Anti-pIKKα  (Ser176/180) | #2697L | 1:1000 | Cell signaling; Shanghai, China |
| Anti-IKKα | #MA5-16157 | 1:1000 | Thermo Fisher |
| Anti-pIκBα  (Ser32, Ser36) | #MA5-15224 | 1:1000 | Thermo Fisher |
| Anti-IκBα | #MA5-16152 | 1:1000 | Thermo Fisher |
| Anti-LSD1 | #PA5-17361 | 1:6000 | Thermo Fisher |
| Anti-GAPDH | #39-8600 | 1:5000 | Thermo Fisher |
| Anti-α-Tubulin | #A11126 | 1:8000 | Thermo Fisher |
| Anti-GST | #MA4-004 | 1:7000 | Thermo Fisher |
| Anti-His | #MA1-21315 | 1:6500 | Thermo Fisher |
| Anti-Flag | #MA1-91878 | 1:7500 | Thermo Fisher |
| Anti-Myc | #PA1-981 | 1:7500 | Thermo Fisher |

GAPDH, Glyceraldehyde 3-phosphate dehydrogenase; IκBα, Inhibitor of kappa B alpha; IKKα, Inhibitory-κB kinase α; LSD1，Lysine-Specific Demethylase 1

**Table S5. The top 20 upregulated genes and top 10 downregulated genes in ARDS lung tissues by RNA-Seq**

| **Genes** | **Control** | **ARDS** |
| --- | --- | --- |
| IL-1B | -4.5 | 4.6 |
| IL-6 | -4.1 | 4.3 |
| IL-15 | -4.1 | 4.2 |
| IL-18 | -4 | 3.7 |
| TNFA | -3.6 | 4.5 |
| IFNG | -3.5 | 4.1 |
| S100A8 | -3.5 | 3.2 |
| S100A9 | -3.5 | 3 |
| SHC1 | -3.4 | 4.2 |
| CPS1 | -3.2 | 4 |
| DNAI1 | -4.3 | 3.2 |
| DNAH3 | -3.7 | 2.6 |
| ARMC3 | -4.3 | 3.2 |
| KEKT4 | -4.1 | 2.8 |
| CDHR4 | -3.5 | 3.5 |
| CtBP2 | -4.6 | 3.8 |
| C3AR1 | -4.1 | 4 |
| ICAM1 | -4.3 | 3.4 |
| RSPO2 | -2.6 | 4.1 |
| CCDC114 | -3.2 | 3.3 |
| SPP1 | 4.1 | -4.5 |
| FBN1 | 4.6 | -4.8 |
| SPSB1 | 4.2 | -4.3 |
| SPSB2 | 4 | -3.6 |
| IBSP | 3.7 | -4.6 |
| HSPB1 | 3.4 | -5 |
| PSMC3 | 3.2 | -4.4 |
| SKP1 | 4.1 | -3.5 |
| NME2 | 3.2 | -4.2 |
| RPS3 | 3.1 | -4 |

**Table S6. The top 7 upregulated genes and top 11 downregulated genes in LPS-challenged Control^KD^, CtBP2^KD^, p300^KD^, and p65^KD^ cells**

| **Genes** | **Control^KD^** | **CtBP2^KD^** | **p300^KD^** | **p65^KD^** |
| --- | --- | --- | --- | --- |
| LCP1 | -4.5 | 4.6 | 5.7 | 7.2 |
| NME2 | -4.1 | 4.3 | 6.8 | 4.1 |
| CSF2 | -6.2 | 7.2 | 6.2 | 4.3 |
| CDH1 | -5.7 | 5.4 | 5.7 | 6.5 |
| SOCS1 | -7.2 | 7.1 | 4.3 | 7.2 |
| IRF1 | -5.5 | 5.6 | 6.7 | 4.6 |
| PRMT5 | -4.6 | 6.8 | 4.5 | 4.1 |
| IL-1B | 8.2 | -7.2 | -4.6 | -6.3 |
| IL-6 | 7.4 | -6.5 | -6.2 | -4.3 |
| IL-18 | 6.4 | -7 | -5.3 | -3.8 |
| IL-15 | 7.4 | -5.7 | -6.7 | -7.2 |
| TNFA | 5.5 | -6.3 | -4.5 | -4.1 |
| IFNG | 7.2 | -6.7 | -6.1 | -4.6 |
| NOX1 | 6.7 | -5.2 | -4.7 | -7.2 |
| BMP2 | 5.3 | -4.5 | -7.2 | -4.1 |
| S100A8 | 4.2 | -5.1 | -6.6 | -6.8 |
| S100A9 | 5.6 | -4.6 | -6.1 | -5.4 |
| CNTN2 | 4.5 | -6.2 | -4.5 | -7.1 |
